# Direct and Absolute Quantification of over 1800 Yeast Proteins via Selected Reaction Monitoring* [Fn FN2]

**DOI:** 10.1074/mcp.M115.054288

**Published:** 2016-01-10

**Authors:** Craig Lawless, Stephen W. Holman, Philip Brownridge, Karin Lanthaler, Victoria M. Harman, Rachel Watkins, Dean E. Hammond, Rebecca L. Miller, Paul F. G. Sims, Christopher M. Grant, Claire E. Eyers, Robert J. Beynon, Simon J. Hubbard

**Affiliations:** From the ‡Faculty of Life Sciences, University of Manchester, Manchester, M13 9PT, UK;; §Centre for Proteome Research, Institute of Integrative Biology, University of Liverpool, Liverpool, L69 7ZB, UK

## Abstract

Defining intracellular protein concentration is critical in molecular systems biology. Although strategies for determining relative protein changes are available, defining robust absolute values in copies per cell has proven significantly more challenging. Here we present a reference data set quantifying over 1800 *Saccharomyces cerevisiae* proteins by direct means using protein-specific stable-isotope labeled internal standards and selected reaction monitoring (SRM) mass spectrometry, far exceeding any previous study. This was achieved by careful design of over 100 QconCAT recombinant proteins as standards, defining 1167 proteins in terms of copies per cell and upper limits on a further 668, with robust CVs routinely less than 20%. The selected reaction monitoring-derived proteome is compared with existing quantitative data sets, highlighting the disparities between methodologies. Coupled with a quantification of the transcriptome by RNA-seq taken from the same cells, these data support revised estimates of several fundamental molecular parameters: a total protein count of ∼100 million molecules-per-cell, a median of ∼1000 proteins-per-transcript, and a linear model of protein translation explaining 70% of the variance in translation rate. This work contributes a “gold-standard” reference yeast proteome (including 532 values based on high quality, dual peptide quantification) that can be widely used in systems models and for other comparative studies.

Reliable and accurate quantification of the proteins present in a cell or tissue remains a major challenge for post-genome scientists. Proteins are the primary functional molecules in biological systems and knowledge of their abundance and dynamics is an important prerequisite to a complete understanding of natural physiological processes, or dysfunction in disease. Accordingly, much effort has been spent in the development of reliable, accurate and sensitive techniques to quantify the cellular proteome, the complement of proteins expressed at a given time under defined conditions (1). Moreover, the ability to model a biological system and thus characterize it in kinetic terms, requires that protein concentrations be defined in absolute numbers (2, 3).

Given the high demand for accurate quantitative proteome data sets, there has been a continual drive to develop methodology to accomplish this, typically using mass spectrometry (MS) as the analytical platform. Many recent studies have highlighted the capabilities of MS to provide good coverage of the proteome at high sensitivity often using yeast as a demonstrator system (4–10), suggesting that quantitative proteomics has now “come of age” (1). However, given that MS is not inherently quantitative, most of the approaches produce *relative* quantitation and do not typically measure the absolute concentrations of individual molecular species by direct means.

For the yeast proteome, epitope tagging studies using green fluorescent protein or tandem affinity purification tags provides an alternative to MS. Here, collections of modified strains are generated that incorporate a detectable, and therefore quantifiable, tag that supports immunoblotting or fluorescence techniques (11, 12). However, such strategies for copies per cell (cpc) quantification rely on genetic manipulation of the host organism and hence do not quantify endogenous, unmodified protein. Similarly, the tagging can alter protein levels - in some instances hindering protein expression completely (11). Even so, epitope tagging methods have been of value to the community, yielding high coverage quantitative data sets for the majority of the yeast proteome (11, 12).

MS-based methods do not rely on such nonendogenous labels, and can reach genome-wide levels of coverage. Accurate estimation of absolute concentrations *i.e.* protein copy number per cell, also usually necessitates the use of (one or more) external or internal standards from which to derive absolute abundance (4). Examples include a comprehensive quantification of the *Leptospira interrogans* proteome that used a 19 protein subset quantified using selected reaction monitoring (SRM)^1^ to calibrate their label-free data (8, 13). It is worth noting that epitope tagging methods, although also absolute, rely on a very limited set of standards for the quantitative western blots and necessitate incorporation of a suitable immunogenic tag (11). Other recent, innovative approaches exploiting total ion signal and internal scaling to estimate protein cellular abundance (10, 14), avoid the use of internal standards, though they do rely on targeted proteomic data to validate their approach.

The use of targeted SRM strategies to derive proteomic calibration standards highlights its advantages in comparison to label-free in terms of accuracy, precision, dynamic range and limit of detection and has gained currency for its reliability and sensitivity (3, 15–17). Indeed, SRM is often referred to as the “gold standard proteomic quantification method,” being particularly well-suited when the proteins to be quantified are known, when appropriate surrogate peptides for protein quantification can be selected *a priori*, and matched with stable isotope-labeled (SIL) standards (18–20). In combination with SIL peptide standards that can be generated through a variety of means (3, 15), SRM can be used to quantify low copy number proteins, reaching down to ∼50 cpc in yeast (5). However, although SRM methodology has been used extensively for *S. cerevisiae* protein quantification by us and others (19, 21, 22), it has not been used for large protein cohorts because of the requirement to generate the large numbers of attendant SIL peptide standards; the largest published data set is only for a few tens of proteins.

It remains a challenge therefore to robustly quantify an entire eukaryotic proteome in *absolute* terms by *direct* means using targeted MS and this is the focus of our present study, the Census Of the Proteome of Yeast (CoPY). We present here direct and absolute quantification of nearly 2000 endogenous proteins from *S. cerevisiae* grown in steady state in a chemostat culture, using the SRM-based QconCAT approach. Although arguably not quantification of the entire proteome, this represents an accurate and rigorous collection of direct yeast protein quantifications, providing a gold-standard data set of endogenous protein levels for future reference and comparative studies. The highly reproducible SIL-SRM MS data, with robust CVs typically less than 20%, is compared with other extant data sets that were obtained *via* alternative analytical strategies. We also report a matched high quality transcriptome from the same cells using RNA-seq, which supports additional calculations including a refined estimate of the total protein content in yeast cells, and a simple linear model of translation explaining 70% of the variance between RNA and protein levels in yeast chemostat cultures. These analyses confirm the validity of our data and approach, which we believe represents a state-of-the-art absolute quantification compendium of a significant proportion of a model eukaryotic proteome.

## EXPERIMENTAL PROCEDURES

### 

#### 

##### Yeast Growth and Sample Preparation

*Saccharomyces cerevisiae* (EUROSCARF accession number Y11335 BY4742; *Mat ALPHA*; *his3*Δ*1*; *leu2*Δ*0*; *lys2*Δ*0*; *ura3*Δ*0; YJL088w::kanMX4*) was grown in defined minimal C-limiting (F1) medium (23) using 10 g/l of glucose as the sole carbon source. The F1 medium was additionally supplemented with 0.5 mm arginine and 1 mm lysine to meet the added auxotrophic requirements of the strain. For biological replication, four cultures were grown in chemostat mode at a dilution rate of 0.1/h and aliquots (15 ml) of the culture were centrifuged (4000 rpm; 4 °C; 10 min). The supernatant was discarded, the pellet flash frozen in liquid nitrogen and stored at −80 °C for subsequent protein extraction. Cell counts were performed using an automated cell counter (Cellometer AUTOM10 by Nexcelom, Lawrence, MA, http://www.nexcelom.com). Proteins were extracted by resuspending the biomass pellets in 250 μl of 50 mm ammonium bicarbonate (filter sterilized) containing 1 tablet of Roche complete-mini protease inhibitors (+ EDTA) (Roche Diagnostics Ltd, West Sussex, UK) per 10 ml of ammonium bicarbonate. Acid washed glass beads (200 μl) were added. The pellet was subjected to repeated bead-beating for 15 bursts of 30 s with a 1 min cool down in between each cycle. The biomass was centrifuged for 10 min at 13,000 rpm at 4 °C; the supernatant was removed and stored in low bind tubes on ice. Fresh ammonium bicarbonate (250 μl) with protease inhibitors was added and the pellet was resuspended by vortex mixing. The bottom of the extraction vial was pierced with a hot needle, the vial placed on a fresh low bind microcentrifuge tube and quickly centrifuged (5 min at 4000 rpm at 4 °C). The flow-through and the supernatant fraction were combined, the exact volume measured and the amount of protein determined by standard Bradford assay (Bio-Rad Laboratories Ltd, Hertfordshire, UK). Protein extracts were aliquoted and stored at −80 °C prior to subsequent digestion.

##### QconCAT Design and Sample Preparation

QconCATs were designed as described previously (2, 19), containing on average 42 Q-peptides acting as surrogate markers for protein quantification. This process included careful selection and ordering of Q-peptides to avoid, where possible, the likelihood of incomplete cleavage in the QconCATs and selection of peptides with poor endogenous cleavage contexts, as estimated by our prediction algorithm McPred (24). A complete list of all 109 QconCATs designed and synthesized along with their Q-peptides and parent proteins is provided in the supplemental Data S1. Proteins targeted for quantification were assembled into the QconCATs, as far as was feasible, by functional groups.

To improve the rigor of quantification and to address the differences in abundance of the native parent proteins within the QconCATs, multiple analytical runs were performed at different loadings of QconCAT in an attempt to constrain analyte/standard ratios between 10:1 and 1:10. To achieve this, three separate yeast digests were performed for each bioreplicate, one of which was spiked with QconCAT to enable codigestion. Yeast lysate representing protein from 21.5 × 10^6^ cells was dispensed into low bind microcentrifuge tubes and made up to 150 μl by addition of 25 mm ammonium bicarbonate, and, in the case of the QconCAT co-digests, 21.6 pmol of QconCAT solution was added. The proteins were denatured by addition of 10 μl of 1% (w/v) RapiGest™ (Waters, Elstree, UK) in 25 mm ammonium bicarbonate and followed by incubation at 80 °C for 10 min. The sample was then reduced (addition of 10 μl of 60 mm DTT and incubation at 60 °C for 10 min) and alkylated (addition of 10 μl of 180 mm iodoacetamide and incubation at room temperature for 30 min in the dark). To allow quantification of the QconCAT, a matched 10 μl of 2.15 pmol/μl glu-fibrinopeptide (Waters) was added to each digest. Trypsin (Sigma, Poole, UK, proteomics grade) was reconstituted in 50 mm acetic acid to a concentration of 0.2 μg/μl and 10 μl added to the sample followed by incubation at 37 °C. After 4.5 h an additional 10 μl of trypsin was added and the digestion left to proceed overnight. The digestion was terminated and RapiGest™ removed by acidification (3 μl of trifluroacetic acid and incubation at 37 °C for 45 min) and centrifugation (15,000 × *g* for 15 min). To check for complete digestion and to quantify the QconCAT, each digest was analyzed by LC-MS using a nanoAcquity UPLC™ system (Waters) coupled to a Synapt™ G2 mass spectrometer (Waters) in MS^E^ mode and searched against a sequence database (See supplementary Methods). The QconCAT was quantified by integrating the peaks generated from the extracted ion chromatogram (XIC) of *m/*z 785.8 (internal standard glu-fibrinopeptide) and *m/z* 788.8 (isotopically labeled glu-fibrinopeptide from QconCAT digestion).

##### SRM Assay Design and Mass Spectrometry

Transitions were selected through the analysis of tryptic digests of the purified QconCATs. Approximately 50–100 fmol of digested QconCAT was loaded onto a nanoAcquity UPLC™ system coupled to a Synapt™ G2 mass spectrometer and product ion spectra acquired in MS^E^ mode. The acquired data was supplemented with extant spectral libraries downloaded from PeptideAtlas (http://www.peptideatlas.org/speclib/) and six transitions per peptide selected. Primarily, transition selection was based on signal intensity, although preference was given to y-ions with *m*/*z* values greater than the precursor ion.

SRM analysis was performed using a nanoAcquity UPLC™ system coupled to a Xevo^TM^ TQMS tandem quadrupole mass spectrometer (Waters). Both quadrupole mass analysers were set to operate at unit mass resolution. To enable time-scheduled acquisition of data, 20 fmol of QconCAT tryptic peptides in a background of 1 μg of yeast tryptic peptides were analyzed on a 60 min LC gradient (3–40% 0.1% formic acid in acetonitrile) to empirically determine the retention times of the Q-peptides. The data was also used to select the three optimal transitions in respect of signal-to-background ratio. From the retention time determination data, time-scheduled methods were constructed using 3 min windows. The methods stipulated the acquisition of 12 data points over a 15 s chromatographic peak width, and each transition had a minimum dwell time of 40 ms typically obtained from two injections. For the final quantification experiment, samples containing the protein equivalent of 200,000 cells and a spike of QconCAT at low (100–250 amol), medium (1–2 fmol) and high (10–20 fmol) concentrations were analyzed. The samples were prepared by serial dilution of the yeast-QconCAT co-digest using a 1:1 mix of the two unspiked yeast digests.

##### Data Processing and FDR Analysis

The mProphet package (25) was used to calculate peptide quantification values from the acquired SRM data, using decoy transitions in order to estimate false discovery rates (FDRs). The decoy transitions were generated using the mGen step of the mProphet pipeline (using the SPIKE_IN workflow option) based on the transitions for the target peptides. The Waters .*raw* files were converted into *mzXML* format using the conversion program wolf-MRM (available at: http://tools.proteomecenter.org/software/wolf-mrm/wolf-mrm.zip). Converted mzXML files were then submitted to the mMap step by setting the –*mach* parameter to *TSQ* and providing the output csv file from mGen. The resulting xml files were then submitted to the mQuest program for peak picking using an optimized parameter file (supporting information). The mQuest xml output was submitted to mProphet to generate the target/reference peptide ratios and associated FDR estimates. Final peptide quantification values, in terms of cpc, were then calculated using the target:reference ratio, known concentration of spike-in heavy QconCAT reference, and the yeast cell count loaded onto the column. In addition, peptide quantification values were only reported when at least three out of four biological replicates passed at a 1% FDR threshold and all had a signal/noise ratio greater than five.

Peptide cpc variance was assessed *via* the robust CV, calculated as 1.4826 times the median absolute deviation: *MAD* = *median*(|*X_i_* − *median_j_*(*X_j_*)|). Protein rCVs were taken directly from peptide rCVs when inferred from a single peptide value, or recalculated using all the peptide values in AA proteins.

##### RNA Extraction, Library Preparation, and Sequencing

RNA extraction using one 15 ml aliquot of the frozen yeast biomass was carried out following previous methods (26). All solutions used were prepared with DEPC (diethylpyrocarbonate 0.1% v/v) treated water. Frozen sample aliquots were ground to a fine powder under liquid nitrogen (26). Pestle and mortar were soaked in 10% bleach to destroy residual RNase activity and washed with diethylpyrocarbonate (DEPC) treated water. RNA was extracted using TriZol® reagent according to the methods of Hayes *et al.*(23) and the final concentration was measured prior to RNA sequencing using a NanoDrop system. Sequencing libraries were generated using the whole Transcriptome Library Preparation protocol provided with the SOLiD® Total RNA-Seq Kit (Life Technologies, Carlsbad, CA). Briefly, rRNA depleted samples were fragmented using RNase III, and subsequently cleaned up using the RiboMinus™ Concentration Modules (Life technologies, Carlsbad, CA). Fragmentation was assessed on a 2100 Bioanalyzer (Agilent Technologies, Palo Alto, CA) using the RNA picochip. Fragmented RNAs were reverse transcribed and size selected on a denaturing polyacrylamide gel selecting for 150–250nt cDNA. cDNA was then amplified and barcoded with SOLiD™ RNA barcoding Kit. Samples were then purified using PureLink™ PCR Micro Kit (Life Technologies) and assessed on a 2100 Bioanalyzer (Agilent Technologies) using the High Sensitivity DNA chip. Samples were deposited on slides, and sequenced using the SOLiD v4 sequencing system (Life Technologies), to an average depth exceeding 4 million reads per library, across four biological replicates.

Reads were mapped to a reference genome of *S. cerevisiae*, downloaded from the Saccharomyces Genome Database (SGD), using Bowtie version 1 (27). Mapped sequences were then assembled into transcripts and quantified using Cufflinks version 2.0 (28) using the SGD reference genome GTF file. Counts were aggregated over the four replicates to generate estimates of transcript abundance expressed as FPKM values for 6581 mRNAs. All data is available from the Gene Expression Omnibus (http://www.ncbi.nlm.nih.gov/geo/) with accession GSE73898, and the FPKM values reproduced in supplemental Data S1.

## RESULTS

**Fig. 1.**
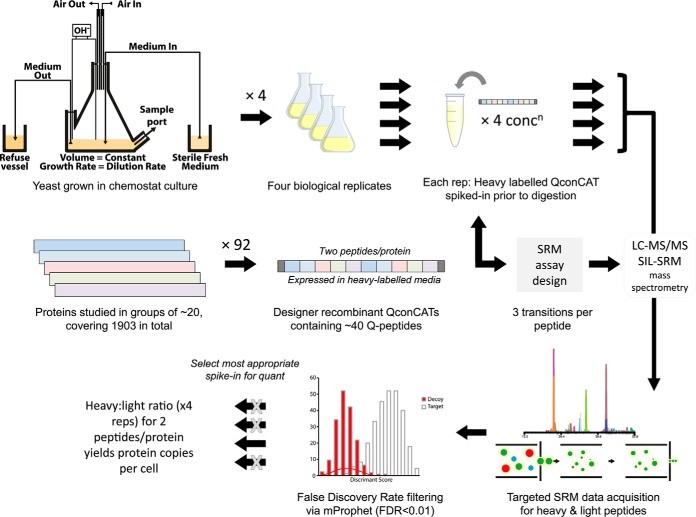
**Schematic overview of QconCAT-based quantification of the yeast proteome using SIL-SRM methodology.** The experimental workflow is depicted in schematic form, showing how chemostat grown yeast samples are extracted, using four biological replicates, for analysis. These samples were combined with designer QconCAT proteins, containing surrogate quantotypic peptides, expressed in a stable-isotope labeled media. SRM assays, designed using a digest of the expressed QconCATs to generate Q-peptides, were then used to quantify the parent proteins. Mixtures of purified QconCAT and yeast proteins were mixed at four concentrations (one of which contained yeast but no QconCAT) and analyzed by SRM-MS to yield SRM chromatogram peak groups for both light (endogenous yeast) and heavy (Q-) peptides. Subsequent quality control by signal:noise cutoffs and mProphet FDR (estimated from decoy transitions) yielded peptide-level copies per cell values, which were then integrated to the protein level for a final quantification.

Our aim was to define the *absolute* concentration of the *Saccharomyces cerevisiae* proteome by direct means, in copies per cell, for cells growing in chemostat culture. Analysis was performed using targeted MS, specifically stable-isotope dilution (SID) SRM-MS, using SIL peptides generated *via* the QconCAT strategy (18, 20). An overview of the workflow is shown conceptually in Fig. 1.

### 

#### 

##### Protein Quantification by QconCAT

Proteins were quantified from the integrated chromatographic peaks described by the SRM-MS data of selected transitions from the predetermined surrogate peptides. These peak areas were calibrated against known spiked-in quantities of heavy isotope-labeled, matched Q-peptides generated from the designed QconCATs, according to the classical isotope dilution MS methodology. This permitted direct absolute quantification of the proteins of interest in cpc, across four biological replicates. Two peptides were nominated to serve as surrogates to quantify each protein, with peptide selection being based on design principles and predictive tools that were developed expressly for this purpose (2, 19, 24, 29). We describe these peptides as “quantotypic,” because they must be both frequently observed under standard experimental conditions (*i.e.* “proteotypic”) and truly quantitative; they should not lose signal because of suboptimal (incomplete) proteolysis, they should not be (or predicted to be) post-translationally modified, and should not be subject to chemical modification, such as oxidation. All of these issues could potentially result in signal splitting leading to sub-stoichiometric amounts compared with their parent protein. These are important considerations when the endogenous protein and labeled standard usually have different proteolytic cleavage contexts. Digestion conditions have been shown to influence subsequent quantitation (30) and some studies have used “spacer” peptides between the Q-peptides that better emulate the native protein's cleavage context, with notable improvements in some cases (31–33). However, when attempting 2000+ proteins the inclusion of spacers was not considered cost-effective, and we simply concatenated native Q-peptides reasoning that if the digestion proceeds to near-completion then the issue of differential cleavage kinetics is not relevant. Furthermore, we used our missed cleavage prediction algorithm (24) to mitigate against the generation of poor cleavage contexts in the QconCATs and avoided selecting peptides with poorly predicted endogenous cleavage sites. Although we recognize that inclusion of natural flanking spacers offers some potential benefits, we believe that a robust single, digestion protocol and careful design offset these concerns, coupled to the consideration of two peptides per protein. This is discussed further in the supplemental Material and Fig. S11.

Despite the extensive design principles, both surrogate peptides did not always yield a detectable SRM signal for either the yeast analyte (light) or, less frequently, for the artificial QconCAT protein-derived standard (heavy). We refer to the quantification outcome according to the nomenclature developed previously (2): Type A, where acceptable data is available for both the native yeast analyte and the isotope-labeled Q-peptides; Type B where the analyte quantotypic peptide was not quantifiable although data was obtained for the QconCAT-derived SIL peptide—this therefore defines a conservative upper limit for analyte quantification; and Type C, where neither of the SRM chromatograms for the native (light) or reference (heavy) peptides yielded signal above the minimum signal-to-noise ratio of five.

To date, we have attempted to quantify a total of 1903 protein groups, from 3835 unique peptides contained within 92 specifically designed QconCAT proteins, yielding 1700 (44.4%) type A, 1476 (38.4%) type B and 659 (17.2%) type C peptides respectively. This equates to a peptide-level success rate of 83% of peptides capable of yielding quantitative information (see supplemental Data S1 and supplemental Fig. S1 for a detailed breakdown of the Q-peptides selected and associated statistics). Peptide quantification was highly repeatable, with a median robust coefficient of variation (rCV) of 11.4% across the replicates (supplemental Fig. 2*B*), which is comparable to or better than similar SRM-based studies (6, 22). Significantly, these studies have yielded a total of 9865 validated yeast SRM transitions for use by the community (supplemental Data S2), which are available from Peptide Atlas via PASSEL (accession PASS00717).

**Fig. 2.**
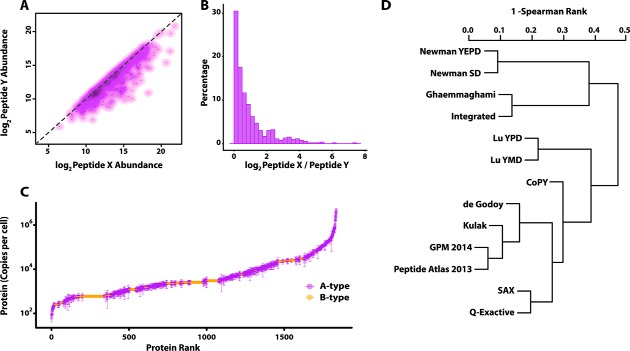
**Peptide and protein level quantification statistics.** *A*, Peptide level abundance (copies per cell) displayed for the 532 matched sibling peptide pairs for Type A proteins, where the paired peptide abundances are shown *X* > *Y* in all cases, as a smoothed scatterplot. The bulk of the points lie on the *x* = *y* line, as shown by the high density of points, though some show deviation from expectation. *B*, Histogram of the log ratios of the sibling peptides (log_2_ *X*/*Y*). The majority of peptides have log2 ratio less than 1, meaning their cpc values are within twofold of each other. *C*, S-curve scatterplot plot of the complete range of protein level cpc values spanning over 4 orders of magnitude, distinguishing A-type from B-type quantification. *D*, Hierarchical clustering dendrogram of independent quantitative proteomes of yeast, based on pairwise Spearman Rank correlations. The various datasets were acquired by different laboratories and by different methods. Data sets were either determined in this study (CoPY, SAX, and Q-Exactive, see Methods) or taken from PaxDb (38). They are associated with the following studies: Ghaemmaghami (11), Newman (12), Lu (39), de Godoy (4), Kulak (10), or from PaxDb directly.

Although more surrogate peptides could potentially improve the accuracy of protein quantification, our choice of two peptides per protein represents a compromise between cost (time and monetary) and analytical rigor. However, such a strategy exposes some of the challenges faced in absolute quantitative proteomics when disagreement arises between the values obtained from sibling peptides. Fortunately, this is relatively rare and good agreement was generally observed between the 532 type A peptide sibling pairs (Fig. 2*A*, 2*B*). Classifying the paired data so that peptide X is always greater than peptide Y, the median log_2_ abundance ratio X/Y for all AA proteins is 0.54; ∼70% of AA proteins have a log_2_ ratio <1 meaning that their peptide cpc values differ by less than twofold. We noted a statistically significant enrichment in certain features of the X and Y peptides in pairs with log_2_ ratios above and below the median (supplemental Fig. S3); most notably, an increased missed cleavage potential in the native protein context of the lower abundance Y peptides (24). Accordingly, we adopted the following protocol for protein level quantification: when the discrepancy between the peptides was less than 0.54, the final protein value was taken as the median average of the two peptide values; for the remaining cases the higher of the two peptides was used for quantification, reasoning that signal loss from endogenous peptide is more likely. For the other classes of protein quantifications (AB and AC) the protein quantification value was taken from the A-class peptide.

We obtained absolute quantification in cpc for 1167 type A proteins (AA, AB, and AC), an upper limit was defined for a further 668 type B proteins (BB, BC), with only 68 type C proteins failing to yield any quantitative information, corresponding to an overall 96% success rate. Formally, the 1167 quantified proteins are protein “groups,” including some homologs that are indistinguishable because of the lack of unique and selectable Q-peptides. Therefore, the 1167 type A quantifications span 1217 yeast ORFs from the genome, covering a wide range of functions (supplemental Fig. S1*B*). For convenience we refer to this as the P1200 dataset of absolute protein quantifications. We also observed good reproducibility at the protein level, with cpc values across biological replicates generating a median rCV of 12.6% (supplemental Fig. S2*C*) with the quantitative values spanning a dynamic range of 5 orders of magnitude from ∼60 cpc (IRS4) to 4.4 × 10^6^ cpc (PDC1), shown in Fig. 2*C*. The dynamic range at the peptide level is shown in supplemental Fig. S2*A*.

Some key points should be emphasized. First, this is the largest direct and absolute quantification of the yeast proteome *via* mass spectrometry obtained to date, with cpc values obtained for endogenous proteins in their native, unmodified form. This distinction is important because most studies define protein changes in relative amounts, or use limited or indirect standards for quantification (11, 34), thereby introducing additional variability. Our approach has internal standards for every peptide. Second, unlike relative quantification studies, absolute data informs on global protein changes, such as those that might be introduced in a mutant strain or under an environmental stress that perturbs translation genome-wide (*e.g.* (35)). Similarly, relative quantification cannot be used to assess the stoichiometry of protein components of complexes. Finally, knowledge of the absolute protein abundance supports an independent estimate of the total protein content in a cell, and can be used to estimate associated properties such as translational efficiency.

##### Comparison of Yeast Proteome Quantification Data Sets

The utility of a “gold standard” yeast strain is obvious; as has been suggested (36), a standard strain whose proteome is accurately quantified can be used as an internal standard itself for absolute quantification of other yeast proteomes, either in label-free or label-mediated SILAC type workflows. Additionally, SRM-based absolute concentrations can be used to calibrate label-free data to achieve essentially complete proteome coverage (8, 37).

We compared the P1200 absolute quantification data set with the yeast data sets available in the PaxDb database (38), including data sets acquired by epitope tagging *via* TAP (11) and GFP (12), label-free spectral counting (39), SILAC MS (4), as well as a recent high coverage label-free data set (10) and two independent label-free acquisitions performed in our own laboratories relying on data-independent acquisition “Hi-3” quantification (34) (see supplemental Methods and Data S1, full raw data available from ProteomeXchange PXD002694). For consistency, we rescaled data sets not yet in PaxDb to parts-per-million (ppm), the preferred unit of PaxDb, assuming 60 million total protein molecules per cell as the total protein constituency (38, 40). Hierarchical clustering of the data reveals clear trends, shown in Fig. 2*D*, grouping sets by virtue of their underlying methodologies and laboratories. Most notably, the epitope tagging methods and MS-based methods cluster independently, as might be expected and as observed previously for smaller data sets (19). Of potentially greater interest, however, is the similarity between quantitative data sets generated by the *same* laboratory on the *same* yeast but under *different* growth conditions, contrasted with reduced similarity between *different* labs on yeast under the *same* (or very similar) growth conditions. This suggests that the natural biological variance observed from growth differences is typically smaller than the technique-based variance introduced by different laboratories, protocols, and analytical methods. This phenomenon is well illustrated by the epitope tagging methods used by Newman and colleagues (12) that are tightly clustered, as are the spectral counting-based quantifications from Lu and colleagues (39), despite the fact that the paired studies are of yeast grown under different nutrient conditions: rich and minimal media. Similar observations have also been reported for transcriptomic data (41). However, there is clear co-clustering between independent quantifications conducted on very similar yeast samples; the two data sets from the Mann laboratory (de Godoy and Kulak data sets), and our identical chemostat cultures quantified using label-free methods (denoted SAX and Q-Exactive in Fig. 2*D*). Our SRM-based direct quantification is a modest outlier, but clusters with all the mass spectrometry based methods and shows the highest correspondence overall with the SAX and Kulak data sets (Spearman correlations of 0.75 and 0.76). We noted similar good correspondence between QconCAT-derived SRM data and label-free data for a small-scale study of glycolytic enzymes (21). This argues that the choice of analytical approach contributes considerable variance when strains/growth conditions are identical.

We also compared our data with a previous targeted study in yeast that quantified 21 proteins via stable-isotope labeled standards (5). Only nine proteins were quantified by both methods, but there was generally good agreement (*r*^2^ = 0.84 comparing log(cpc)), across this limited data set.

Although these independent studies have used different methods, growth conditions and yeast strains (though generally in the BY background), the correspondence across different datasets is modest (supplemental Fig. S4) with the Spearman rank correlations between different laboratories around ∼0.6–0.7. This is only slightly higher than that typically observed between the proteome and transcriptome within the same organism (42), and close to that observed across species boundaries for the quantitative proteome (43, 44). These results match a recent reanalysis of diverse yeast transcriptome and proteome data sets (45), and reinforce the need for a true gold standard absolute quantification of the yeast proteome (36).

Importantly, the present study quantified proteins that had not been measured previously, whether by antibody-based or other mass spectrometry-based methods (supplemental Fig. S5*C*). We also note that although we obtain A-type quantification as low as ∼50 cpc, the very low abundance B-type proteins (where only an upper limit is defined) correspond to genes with equally low abundance transcripts. These proteins are generally refractive to all quantitative methods but do possess Q-peptides that are equally well predicted to be proteotypic compared with A-type peptides. This contrasts with the C-type peptides that are the poorest predicted and were often selected because no better peptide could be nominated (supplemental Fig. *S*5).

**Fig. 3.**
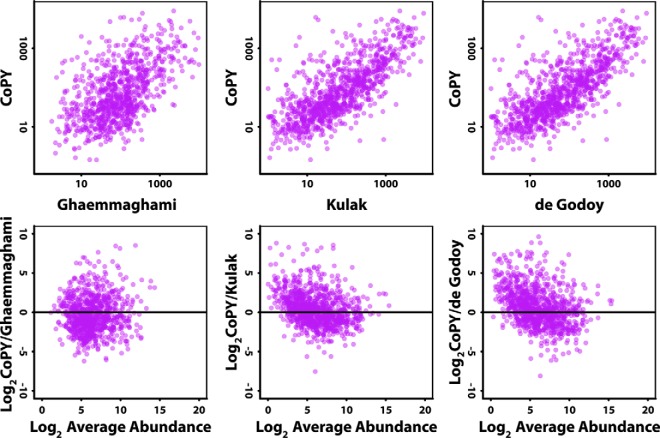
**Example correlation and M-*versus*-A plots for protein abundances from different studies compared with the CoPY project.** Scatterplots showing the correlation between CoPY protein abundance in cpc converted to ppm (assuming 60 million copies per cell) compared with exemplar datasets taken from the PaxDb database. Panel *A–C* show correlation plots for an epitope-tagging method, Ghaemmaghami (11), and a SILAC-based study, de Godoy (4), and a label-free study, Kulak (10). These are matched by M-*versus*-A plots below in *D–F*, calculated by plotting the log ratio of the protein abundances against the average protein abundance. The plots show a systematic trend toward higher protein abundance estimates in the CoPY data for low abundance proteins in the shotgun mass spectrometry studies (*E* and *F*).

A more detailed comparison of the quantification values for contrasting methodological approaches allowed systematic differences to be assessed. Representative scatterplots and “M *versus* A” plots are shown in Fig. 3 for protein values in common between paired approaches, calculated from protein abundances scaled to 60 million copies per cell (see also supplemental Figs. S6 and S7). In comparison with our SRM targeted proteomics approach, epitope tagging methods show reasonable agreement but there is clearly considerable variance across the abundance range. Better agreement is generally observed with mass spectrometry-based methods particularly for proteins at high abundance. We also note a systematic difference between label-free/SILAC MS methods and our targeted SID-SRM approach where proteins of low average abundance are generally determined to be of higher abundance from the SRM experiments. This effect was noted when comparing the targeted data to all label-free approaches and suspect this is because of the systematic under-representation of ions from low abundance proteins in shotgun DDA experiments that leads to underestimation of either spectral counts or ion intensity aggregated to the protein level. Despite this, modern MS instruments are clearly able to offer excellent coverage of the low abundance proteome, down to the tens of copies of proteins per cell (7, 10). It remains to be seen whether equivalent levels of sensitivity can be developed in much larger eukaryotic cells that could contain 50 times as much protein as a yeast cell.

##### Protein Stoichiometry and Abundance in Signaling Modules

Unlike differential expression studies, absolute quantification supports examination of protein stoichiometry and the comparison of different components in a complex, network or pathway. As an exemplar, we considered protein stoichiometry in the anaphase promoting complex/cyclosome (APC/C), a highly regulated cell cycle ubiquitin E3 ligase complex important for entry into S-phase and essential for progression through mitosis and meiosis. Our SID-SRM data did not offer universal coverage of all the proteins involved in this complex; the Apc1 core protein and the anaphase-promoting complex subunit Cdc23 were measured at 260 and 830 cpc, with four of the other core proteins (Apc4, Apc5, Apc10, Apc11) present at <500 cpc and Apc9 at <130 cpc (supplemental Table S1 and Material). This is consistent with previous structural studies, which estimated the relative subunit stoichiometry of Cdc23 to be double that of Apc1/4/5/10/11 using a purified, reconstituted APC/C system (46), and in agreement with the potential additional roles of Cdc23 suggested by its known cellular interactions.

Absolute protein abundance is also relevant to modeling metabolic and regulatory pathways (3, 21). Here, we consider our data in the context of MAP kinase signaling cascades that sensitively propagate signal from the cell surface *via* intracellular effector molecules to elicit a transcriptional response. Because protein kinases, as opposed to protein phosphatases, are thought to be the key regulatory factors in modulating signal amplitude (47), measuring their absolute protein abundance has high value for rationalizing signal amplification. However, to date, most studies have focused on relative quantification of specific phosphopeptide stoichiometries (which could be used as a read out of enzymatic activity) and not the absolute protein levels (c.f (48–50).). The ratio of active enzyme to total available protein dictates whether a pathway becomes “weakly” or “highly” activated and controls the degree of ultrasensitivity of the system. Under normal physiological conditions, most signaling pathways are likely to exist in a weakly activated state, permitting both finer control (shorter signal duration) and the ability to respond rapidly to pathophysiological conditions.

**Fig. 4.**
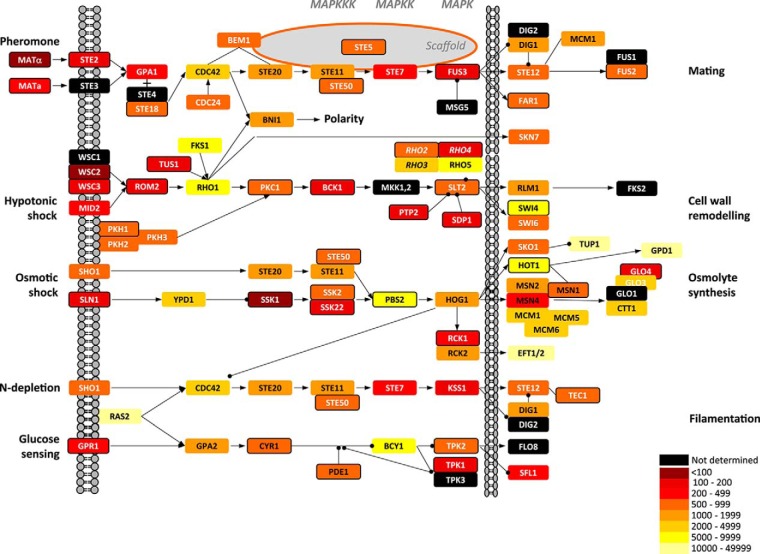
**Protein abundances from the CoPY project mapped to MAP kinase signaling pathways.** Proteins are shown as rectangles, colored by abundance as shown in the key. Despite no single, consistent trend it is apparent that there is not a systematic increase in protein abundance throughout the MAPK pathways as signal is propagated toward the nucleus.

Absolute protein quantification of components of the different MAPK cascades in *S. cerevisiae* shows that, unlike relative enzyme activity, protein amount does not increase uniformly along the pathway (Fig. 4). For example, the Kss1 and Fus3 pathways, which together mediate the responses to mating and filamentation, exhibit a *decrease* in absolute protein levels from Cdc42 through Ste20, to the MAPKKK Ste11, the MAPKK Ste7 and the MAPKs Kss1 and Fus3. However, the effector transcription factors are present at much *higher* numbers. Protein quantity at the different “levels” through the other MAPK pathways (*e.g.* Gpr1 mediated response to glucose sensing) is variable. Rationalization of the differences in protein abundance throughout these cascades is further complicated by extensive cross talk, as kinases frequently regulate the function of multiple substrates (*e.g.* Cdc42 acting on Ste20, Bni1) and thus affect multiple outcomes (*e.g.* polarity, mating, filamentation in fission yeast). Equally, our cpc measurements determine global protein levels and do not reflect any localized protein concentration that may arise as a result of compartmentalization or targeted localization (*e.g.* by attachment to a scaffold such as Ste5). However, coupled to enzymatic assays and phosphopeptide analysis, absolute quantification greatly informs attempts to understand sensitivity and control of signaling (and metabolic) systems.

##### Translational Efficiency of Yeast Gene Expression

**Fig. 5.**
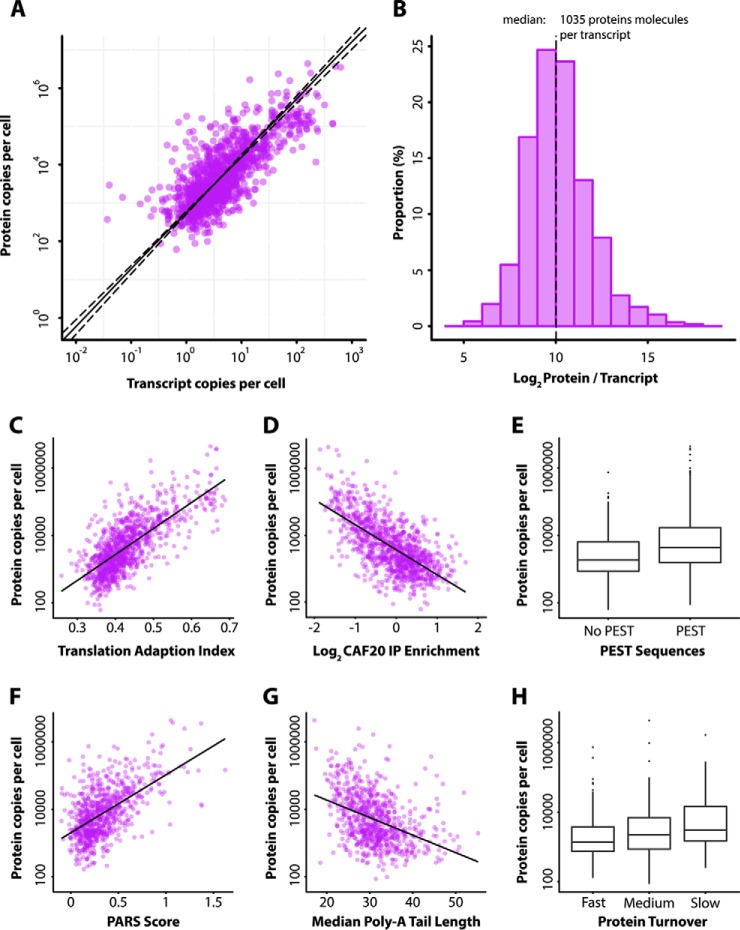
**Translational efficiency and the relationship between transcriptome and proteome.** *A*, Scatterplot showing the relationship between the quantitative proteome and transcriptome in this study for the P1200 set proteins, plotting absolute cpc values matched to the mRNA equivalent derived from their FPKM values. *B*, Histogram of the log_2_ ratio distribution of protein to transcript, for all P1200 set proteins, with median value of 1035 proteins per transcript. Panels *C–H* illustrate the relationship between absolute protein abundance and a subset of the features considered in the linear model construction. *C*, The translational adaptation index (tAI) (68) calculated from P1200 set transcripts show a positive correlation with the respective log protein abundances (*r*^2^ = 0.53, *p* < 2.2 × 10^−16^). *D*, RNA-binding protein immunoprecipitation enrichment of the transcripts for the translation factor CAF20 (67) shows a strong negative relationship to respective log protein abundances (*r*^2^ = 0.42, *p* < 2.2 × 10^−16^). *E*, Boxplots showing a significant but surprising increase in the abundance of the P1200 subset that contain degradative PEST sequences (Wilcoxon rank test, *p* = 2.8 × 10^−12^). *F*, A positive linear correlation (*r*^2^ = 0.33, *p* < 2.2 × 10^−16^) between protein abundance and the transcript propensity to form secondary structure, the PARS score (71). *G*, A weak negative correlation between the median transcript poly-A tail length and protein abundance (*r*^2^ = 0.16, *p* < 2.2 × 10^−16^). *H*, Boxplots of protein abundance values (log scale) for proteins classified into three groups based on measured protein turnover data (66). Groups were defined by protein half-life, 0–20 min (Fast), 20–40 min (Medium) and 40–60 min (Slow). All comparisons of Fast-Medium, Fast-Slow and Medium-Slow show a significant increase in protein abundance using a Wilcoxon rank test with *p* < 0.05.

Akin to previously published studies (4, 11, 39, 51, 52), we quantified the transcriptome of our chemostat grown yeast to compare directly with the proteome. Previous large-scale studies in *S. cerevisiae* have compared relative changes between transcript and protein ratios between cell types (4) and conditions (52), have compared proteome and transcriptome data sets from different studies/conditions (11), or combined multiple proteomic and transcriptomic data sets to produce a reference data set (39). As pointed out by a recent modeling study (45), few have compared high quality matched transcriptome and proteome data from the same yeast cells. Here, we used replicated next-generation sequencing (RNA-seq) to obtain a measure of transcriptome abundance, extrapolating our FPKM values to an estimated mRNA cpc assuming the average yeast cell contains 60,000 total mRNA copies (53). We caution that this is an estimate because we did not directly quantify the absolute transcriptome ourselves, though similar approaches have been taken by other groups (39, 53). A strong and significant correlation was observed between our P1200 protein cpc values and their respective transcript cpc (*r*^2^ = 0.58 and *r*_sp_ = 0.73; Fig. 5*A*). This is in good general agreement with previous estimates (39, 54), but toward the top end of the range of estimated proteome variance explained by the transcriptome (∼60%). This relationship has been a topic of recent debate in the field, with a number of recent studies arguing that the role of post-transcriptional control has been overestimated and the true correlation is closer to 90% (45, 55). This disagreement in the field stems in part from issues with experimental noise, incomplete coverage, and modest experimental repeatability.

As previously reported (39, 54) we also observe a log normal distribution of individual protein/mRNA ratios (Fig. 5*B*), with a higher dynamic range observed in the proteome data (from <10^2^ to >10^6^ copies per cell). These ratios represent the translational efficiencies of individual genes estimated from our direct measurements of absolute protein and mRNA abundance. Our ratios range from the GATA zinc finger protein GZF3 at 40 proteins per transcript, through to ILV6, the regulatory subunit of acetolactate synthase complex, at ∼180,000. The median of 10^3^ protein molecules per transcript is considerably smaller than previous estimates that report values between 4000–5600 (11, 39, 56). We ascribe this in part to our use of the more up-to-date estimate of 60,000 mRNAs in the yeast cell (53) compared with the previously widely reported 15,000 copies per cell. The latter leads to similar protein:RNA estimates with our proteome data of ∼4000 proteins/mRNA. It may also reflect improvements in the underlying technologies used to measure both mRNA and absolute protein copy number, as well as slower growth rates in chemostat cell cultures compared with batch/rich media (57), the latter being used in some previous studies.

Our comprehensive transcriptome data also supports a revised estimate of total protein present in a yeast cell. Because our quantification of 1167 proteins sums to 54 million cpc, we can extrapolate using the median protein copies per transcript, to calculate an estimated sum of about 100 million cpc for the entire proteome. Again, this estimate is larger than previously reported (11, 21, 56), though consistent with recent re-analyses (40) and observations of higher biomass yield in chemostat cultures compared with batch or slower growth rates (57, 58).

The wide range in observed correlations between measured quantitative yeast proteomes and transcriptomes (4, 11, 45, 59, 60) reveals a large variation in the protein/mRNA relationships (45), stressing the importance of paired data from the same cells. Indeed, our quantitative proteome is more tightly clustered with our own transcriptome data set than all other quantitative proteomes (supplemental Fig. S8), reinforcing the necessity to avoid bias where possible by integrating different 'omics data from different labs. A similar observation has been made for RNA-seq platforms (41, 61). We also note that proteo-transcriptome correlations from matched cells in other organisms yield similar values to ours (13, 54), including a recent study in *Schizosaccharomyces pombe* whereby a comparable correlation (*r*^2^ = 0.55) was observed in proliferating cells using protein measurements from label-free MS and transcript measurements from RNA-seq (13).

##### Modeling the Relationship Between Transcriptome and Proteome

Although recognizing that our quantitative data is still subject to experimental noise and modest coverage, we built a simple linear modeling to examine the relationship between mRNA and protein (62–64) and consider the effect of post-transcriptional processes. A linear regression model based on transcript measurements was used to develop a multivariate linear model involving additional translation-associated metrics (60, 65–72) (see Supplemental Methods). The relationship of some of these characteristics to our absolute protein abundance measurements are presented in Figs. 5*C*-5*H*, and the complete list of features assessed in the model are listed in supplemental Table S2. For example, when we classified proteins into three categories of turnover (66); “slow” (half-life < 20 min), “medium” (half-life 20 to 40 min), and “fast” (>40 min), we observe significant differences between the distributions of protein abundance (Wilcoxon rank test, *p* < 0.05, Fig. 4*H*) suggesting it would be informative in the model.

Using an iterative, sequential approach we derived a high performing, multivariate regression model using seven features, three of which were included when used as an interaction term with transcript abundance (see supplemental Methods). The model achieved an *r*^2^ of 0.7 (*p* < 2.2 × 10^−16^) and resulted in a Spearman Rank of 0.83 (*p* < 2.2 × 10^−16^) between real and predicted protein abundances (supplemental Fig. S9). The most significant contributor to the final model (after transcript abundance itself) was the translation adaptation index (tAI), a measure of codon bias, which increases the *r*^2^ from 0.58 to 0.63 (*r*_sp_ of 0.77 from 0.73, *p* < 2.2 × 10^−16^). Although this indicates a positive role for post-transcriptional regulation, the overall increase in the variance explained is modest; 30% of the variance still remains unexplained. As has been recently suggested (45), this could be a result of limitations in the extant data or model, or possibly because of some hitherto unknown control step in translational regulation. Importantly, in this regard, our model is the first large-scale attempt to explain translational control in *S. cerevisiae* using matched mRNA and direct SRM-based protein measurements from the same cells. However, this condition is unfortunately not met by some of the other postgenomic data used in the model, which are derived from different laboratories using different yeast cells grown under different conditions, adding additional noise.

Such experimental noise has been suggested as the principal reason for the apparent disparity between transcriptome and proteome abundance data by Csardi and colleagues (45). They subsequently proposed a simple correction based on the work of Spearman, which uses the inherent repeatability of the individual experimental approaches estimated from biological replicates. Applying the same approach to our data transforms our uncorrected Pearson correlation of 0.72 to *r^corrected^* of 0.74. This modest increase can be attributed to the high repeatability between our replicates; 0.98 and 0.96, for protein and transcript levels respectively. Despite this, our correlation may well still be an underestimate; our data, although comprehensive, is still an under-sampling of the complete proteome and like other approaches has a modest bias against very low abundance proteins. We also considered the log-log correlation between transcriptome and proteome, observing a near unitary slope of 1.08 for ordinary least squares fitting, but a slope of 1.50 following the Ranged Major-Axis approach (supplemental Fig. S10). This is supportive of the assertion that proteome abundance is nonlinearly dependent on the transcriptome (45).

## CONCLUSIONS

We present here the most comprehensive and robust direct absolute quantification of the yeast proteome to date; for nearly 1200 proteins abundance is defined in copies per cell and an upper limit provided for a further 668 proteins. Absolute quantification is of great utility for systems biologists wishing to understand translational control or build kinetic models, to inform on protein stoichiometry by measuring the total cellular abundance of the complex components, and to determine absolute levels distributed throughout regulatory and metabolic pathways. These studies further highlight the value of targeted SRM-based quantification using stable-isotope mediated standards to directly quantify protein abundances. Our careful peptide selection and subsequent design of suitable transitions have added a total of 9865 validated SRMs for community use. Good reproducibility is observed across biological replicates (median rCVs ∼ 13%), as well as very good agreement overall between sibling peptides.

The value of this robust, absolute quantification is demonstrated; offering revised and improved estimates of the total protein copy number in a chemostat grown yeast culture, and associated translational efficiency measures derived from matched RNA-seq data. In turn, the transcriptome data have supported the derivation of an improved model of translation in steady state. We also demonstrate the data's utility to better understand the stoichiometry of molecular machines (APC/C) and signaling pathways (MAPK), which is essential to rationalize their complex biological function.

Although the entire proteome has not been used to quantified via SIL-SRM, we believe the data and yeast strain itself are of high value. The complete QconCAT designs are available for use by other laboratories (see supplemental Data S1 and Data S3), with validated transitions for proven quantotypic peptides, all deposited in the PASSEL database (accession PASS00717) where users can browse the entire collection and examine chromatograms for individual transitions. Indeed, we believe we have more than sufficient quantitative data to complete the comprehensive absolute quantification of the yeast proteome to define a gold standard, exploiting the SRM-derived data to calibrate label-free approaches proteome-wide (7), a strategy that has shown promise with considerably fewer proteins as calibrants (8, 13). Once this has been achieved, the yeast strain described here, if cultured under the same conditions, can act as an internal standard (with or without stable-isotope labeling) for other conditions, strains, and environments, offering a genome-wide calibration set to facilitate routine absolute quantification of the yeast proteome.

## Supplementary Material

Supplemental Data

## Footnotes

^1^ The abbreviations used are:

SRM: selected reaction monitoring
CoPY: Census of the Proteome of Yeast
cpc: copies per cell
FDR: false discovery rate
FPKM: fragments per kilobase of transcript per million mapped reads
PTMs: post-translational modifications
Q-peptides: quantotypic peptides
QconCAT: quantification concatamer
rCV: robust coefficient of variation
SGD: Saccharomyces genome database
SIL: stable-isotope labeled
SWATH: sequential window acquisition of all theoretical mass spectra
TAP: tandem affinity purification.

## References

[1] Mann M., Kulak N. A., Nagaraj N., and Cox J. (2013) The coming age of complete, accurate, and ubiquitous proteomes. Mol. Cell 49, 583–590 23438854 10.1016/j.molcel.2013.01.029

[2] Brownridge P., Holman S. W., Gaskell S. J., Grant C. M., Harman V. M., Hubbard S. J., Lanthaler K., Lawless C., O'Cualain R., Sims P., Watkins R., and Beynon R. J. (2011) Global absolute quantification of a proteome: Challenges in the deployment of a QconCAT strategy. Proteomics 11, 2957–2970 21710569 10.1002/pmic.201100039

[3] Ludwig C., and Aebersold R. (2014) Getting absolute: determining absolute protein quantitites via selected reaction monitoring mass spectrometry. In: Eyers C. E., and Gaskell S. J., eds. Quantitative Proteomics, pp. 80–109, Royal Society of Chemistry, Cambridge

[4] de Godoy L. M., Olsen J. V., Cox J., Nielsen M. L., Hubner N. C., Frohlich F., Walther T. C., and Mann M. (2008) Comprehensive mass-spectrometry-based proteome quantification of haploid versus diploid yeast. Nature 455, 1251–1254 18820680 10.1038/nature07341

[5] Picotti P., Bodenmiller B., Mueller L. N., Domon B., and Aebersold R. (2009) Full dynamic range proteome analysis of S. cerevisiae by targeted proteomics. Cell 138, 795–806 19664813 10.1016/j.cell.2009.05.051 PMC2825542

[6] Picotti P., Clement-Ziza M., Lam H., Campbell D. S., Schmidt A., Deutsch E. W., Rost H., Sun Z., Rinner O., Reiter L., Shen Q., Michaelson J. J., Frei A., Alberti S., Kusebauch U., Wollscheid B., Moritz R. L., Beyer A., and Aebersold R. (2013) A complete mass-spectrometric map of the yeast proteome applied to quantitative trait analysis. Nature 494, 266–270 23334424 10.1038/nature11835 PMC3951219

[7] Hebert A. S., Richards A. L., Bailey D. J., Ulbrich A., Coughlin E. E., Westphall M. S., and Coon J. J. (2014) The one hour yeast proteome. Mol. Cell. Proteomics 13, 339–347 24143002 10.1074/mcp.M113.034769 PMC3879625

[8] Malmstrom J., Beck M., Schmidt A., Lange V., Deutsch E. W., and Aebersold R. (2009) Proteome-wide cellular protein concentrations of the human pathogen Leptospira interrogans. Nature 460, 762–765 19606093 10.1038/nature08184 PMC2723184

[9] Nagaraj N., Kulak N. A., Cox J., Neuhauser N., Mayr K., Hoerning O., Vorm O., and Mann M. (2012) System-wide perturbation analysis with nearly complete coverage of the yeast proteome by single-shot ultra HPLC runs on a bench top Orbitrap. Mol. Cell. Proteomics 11, M111 013722 22021278 10.1074/mcp.M111.013722 PMC3316726

[10] Kulak N. A., Pichler G., Paron I., Nagaraj N., and Mann M. (2014) Minimal, encapsulated proteomic-sample processing applied to copy-number estimation in eukaryotic cells. Nat. Methods 11, 319–324 24487582 10.1038/nmeth.2834

[11] Ghaemmaghami S., Huh W. K., Bower K., Howson R. W., Belle A., Dephoure N., O'Shea E. K., and Weissman J. S. (2003) Global analysis of protein expression in yeast. Nature 425, 737–741 14562106 10.1038/nature02046

[12] Newman J. R., Ghaemmaghami S., Ihmels J., Breslow D. K., Noble M., DeRisi J. L., and Weissman J. S. (2006) Single-cell proteomic analysis of S. cerevisiae reveals the architecture of biological noise. Nature 441, 840–846 16699522 10.1038/nature04785

[13] Marguerat S., Schmidt A., Codlin S., Chen W., Aebersold R., and Bähler J. (2012) Quantitative Analysis of Fission Yeast Transcriptomes and Proteomes in Proliferating and Quiescent Cells. Cell 151, 671–683 23101633 10.1016/j.cell.2012.09.019 PMC3482660

[14] Wisniewski J. R., Hein M. Y., Cox J., and Mann M. (2014) A “Proteomic Ruler” for Protein Copy Number and Concentration Estimation without Spike-in Standards. Mol. Cell. Proteomics 13, 3497–3506 25225357 10.1074/mcp.M113.037309 PMC4256500

[15] Holman S. W., Sims P. F. G., and Eyers C. E. (2012) The use of selected reaction monitoring in quantitative proteomics. Bioanalysis 4, 1763–1786 22877222 10.4155/bio.12.126

[16] Ludwig C., Claassen M., Schmidt A., and Aebersold R. (2012) Estimation of absolute protein quantities of unlabeled samples by selected reaction monitoring mass spectrometry. Mol. Cell. Proteomics 11, M111 013987 22101334 10.1074/mcp.M111.013987 PMC3316728

[17] Picotti P., and Aebersold R. (2012) Selected reaction monitoring-based proteomics: workflows, potential, pitfalls and future directions. Nat. Methods 9, 555–566 22669653 10.1038/nmeth.2015

[18] Beynon R. J., Doherty M. K., Pratt J. M., and Gaskell S. J. (2005) Multiplexed absolute quantification in proteomics using artificial QCAT proteins of concatenated signature peptides. Nat. Methods 2, 587–589 16094383 10.1038/nmeth774

[19] Brownridge P., Lawless C., Payapilly A. B., Lanthaler K., Holman S. W., Harman V. M., Grant C. M., Beynon R. J., and Hubbard S. J. (2013) Quantitative analysis of chaperone network throughput in budding yeast. Proteomics 13, 1276–1291 23420633 10.1002/pmic.201200412 PMC3791555

[20] Pratt J. M., Simpson D. M., Doherty M. K., Rivers J., Gaskell S. J., and Beynon R. J. (2006) Multiplexed absolute quantification for proteomics using concatenated signature peptides encoded by QconCAT genes. Nat. Protoc. 1, 1029–1043 17406340 10.1038/nprot.2006.129

[21] Carroll K. M., Simpson D. M., Eyers C. E., Knight C. G., Brownridge P., Dunn W. B., Winder C. L., Lanthaler K., Pir P., Malys N., Kell D. B., Oliver S. G., Gaskell S. J., and Beynon R. J. (2011) Absolute quantification of the glycolytic pathway in yeast: deployment of a complete QconCAT approach. Mol. Cell. Proteomics 10, M111 007633 21931151 10.1074/mcp.M111.007633 PMC3237070

[22] Mirzaei H., Knijnenburg T. A., Kim B., Robinson M., Picotti P., Carter G. W., Li S., Dilworth D. J., Eng J. K., Aitchison J. D., Shmulevich I., Galitski T., Aebersold R., and Ranish J. (2013) Systematic measurement of transcription factor-DNA interactions by targeted mass spectrometry identifies candidate gene regulatory proteins. Proc. Natl. Acad. Sci. U. S. A. 110, 3645–3650 23388641 10.1073/pnas.1216918110 PMC3587231

[23] Hayes A., Zhang N., Wu J., Butler P. R., Hauser N. C., Hoheisel J. D., Lim F. L., Sharrocks A. D., and Oliver S. G. (2002) Hybridization array technology coupled with chemostat culture: Tools to interrogate gene expression in Saccharomyces cerevisiae. Methods 26, 281–290 12054884 10.1016/S1046-2023(02)00032-4

[24] Lawless C., and Hubbard S. J. (2012) Prediction of missed proteolytic cleavages for the selection of surrogate peptides for quantitative proteomics. Omics 16, 449–456 22804685 10.1089/omi.2011.0156 PMC3437044

[25] Reiter L., Rinner O., Picotti P., Huttenhain R., Beck M., Brusniak M. Y., Hengartner M. O., and Aebersold R. (2011) mProphet: automated data processing and statistical validation for large-scale SRM experiments. Nat. Methods 8, 430–435 21423193 10.1038/nmeth.1584

[26] Sims A. H., Robson G. D., Hoyle D. C., Oliver S. G., Turner G., Prade R. A., Russell H. H., Dunn-Coleman N. S., and Gent M. E. (2004) Use of expressed sequence tag analysis and cDNA microarrays of the filamentous fungus Aspergillus nidulans. Fungal Genetics Biol. 41, 199–212 14732266 10.1016/j.fgb.2003.11.005

[27] Langmead B., Trapnell C., Pop M., and Salzberg S. L. (2009) Ultrafast and memory-efficient alignment of short DNA sequences to the human genome. Genome Biol. 10, R25 19261174 10.1186/gb-2009-10-3-r25 PMC2690996

[28] Trapnell C., Williams B. A., Pertea G., Mortazavi A., Kwan G., van Baren M. J., Salzberg S. L., Wold B. J., and Pachter L. (2010) Transcript assembly and quantification by RNA-Seq reveals unannotated transcripts and isoform switching during cell differentiation. Nat. Biotech. 28, 511–515 10.1038/nbt.1621 PMC3146043 20436464

[29] Eyers C. E., Lawless C., Wedge D. C., Lau K. W., Gaskell S. J., and Hubbard S. J. (2011) CONSeQuence: prediction of reference peptides for absolute quantitative proteomics using consensus machine learning approaches. Mol. Cell. Proteomics 10, M110 003384 21813416 10.1074/mcp.M110.003384 PMC3226394

[30] Lowenthal M. S., Liang Y., Phinney K. W., and Stein S. E. (2014) Quantitative bottom-up proteomics depends on digestion conditions. Anal. Chem. 86, 551–558 24294946 10.1021/ac4027274

[31] Scott K. B., Turko I. V., and Phinney K. W. (2015) Quantitative performance of internal standard platforms for absolute protein quantification using multiple reaction monitoring-mass spectrometry. Anal. Chem. 87, 4429–4435 25812027 10.1021/acs.analchem.5b00331

[32] Cheung C. S., Anderson K. W., Wang M., and Turko I. V. (2015) Natural flanking sequences for peptides included in a quantification concatamer internal standard. Anal. Chem. 87, 1097–1102 25522095 10.1021/ac503697j

[33] Chen J. J., and Turko I. V. (2014) Trends in QconCATs for targeted proteomics. Trac-Trend Anal. Chem. 57, 1–5

[34] Silva J. C., Gorenstein M. V., Li G. Z., Vissers J. P., and Geromanos S. J. (2006) Absolute quantification of proteins by LCMSE: a virtue of parallel MS acquisition. Mol. Cell. Proteomics 5, 144–156 16219938 10.1074/mcp.M500230-MCP200

[35] Shenton D., Smirnova J. B., Selley J. N., Carroll K., Hubbard S. J., Pavitt G. D., Ashe M. P., and Grant C. M. (2006) Global translational responses to oxidative stress impact upon multiple levels of protein synthesis. J. Biol. Chem. 281, 29011–29021 16849329 10.1074/jbc.M601545200

[36] Paulovich A. G., Billheimer D., Ham A. J., Vega-Montoto L., Rudnick P. A., Tabb D. L., Wang P., Blackman R. K., Bunk D. M., Cardasis H. L., Clauser K. R., Kinsinger C. R., Schilling B., Tegeler T. J., Variyath A. M., Wang M., Whiteaker J. R., Zimmerman L. J., Fenyo D., Carr S. A., Fisher S. J., Gibson B. W., Mesri M., Neubert T. A., Regnier F. E., Rodriguez H., Spiegelman C., Stein S. E., Tempst P., and Liebler D. C. (2010) Interlaboratory study characterizing a yeast performance standard for benchmarking LC-MS platform performance. Mol. Cell. Proteomics 9, 242–254 19858499 10.1074/mcp.M900222-MCP200 PMC2830837

[37] Gerster S., Kwon T., Ludwig C., Matondo M., Vogel C., Marcotte E. M., Aebersold R., and Buhlmann P. (2014) Statistical approach to protein quantification. Mol. Cell. Proteomics 13, 666–677 24255132 10.1074/mcp.M112.025445 PMC3916661

[38] Wang M., Weiss M., Simonovic M., Haertinger G., Schrimpf S. P., Hengartner M. O., and von Mering C. (2012) PaxDb, a database of protein abundance averages across all three domains of life. Mol. Cell. Proteomics 11, 492–500 22535208 10.1074/mcp.O111.014704 PMC3412977

[39] Lu P., Vogel C., Wang R., Yao X., and Marcotte E. M. (2007) Absolute protein expression profiling estimates the relative contributions of transcriptional and translational regulation. Nat. Biotechnol. 25, 117–124 17187058 10.1038/nbt1270

[40] Milo R. (2013) What is the total number of protein molecules per cell volume? A call to rethink some published values. Bioessays 35, 1050–1055 24114984 10.1002/bies.201300066 PMC3910158

[41] Li S., Tighe S. W., Nicolet C. M., Grove D., Levy S., Farmerie W., Viale A., Wright C., Schweitzer P. A., Gao Y., Kim D., Boland J., Hicks B., Kim R., Chhangawala S., Jafari N., Raghavachari N., Gandara J., Garcia-Reyero N., Hendrickson C., Roberson D., Rosenfeld J., Smith T., Underwood J. G., Wang M., Zumbo P., Baldwin D. A., Grills G. S., and Mason C. E. (2014) Multi-platform assessment of transcriptome profiling using RNA-seq in the ABRF next-generation sequencing study. Nat. Biotechnol. 32, 915–925 25150835 10.1038/nbt.2972 PMC4167418

[42] Vogel C., and Marcotte E. M. (2012) Insights into the regulation of protein abundance from proteomic and transcriptomic analyses. Nat. Rev. Genet. 13, 227–232 22411467 10.1038/nrg3185 PMC3654667

[43] Laurent J. M., Vogel C., Kwon T., Craig S. A., Boutz D. R., Huse H. K., Nozue K., Walia H., Whiteley M., Ronald P. C., and Marcotte E. M. (2010) Protein abundances are more conserved than mRNA abundances across diverse taxa. Proteomics 10, 4209–4212 21089048 10.1002/pmic.201000327 PMC3113407

[44] Weiss M., Schrimpf S., Hengartner M. O., Lercher M. J., and von Mering C. (2010) Shotgun proteomics data from multiple organisms reveals remarkable quantitative conservation of the eukaryotic core proteome. Proteomics 10, 1297–1306 20077411 10.1002/pmic.200900414

[45] Csardi G., Franks A., Choi D. S., Airoldi E. M., and Drummond D. A. (2015) Accounting for experimental noise reveals that mRNA levels, amplified by post-transcriptional processes, largely determine steady-state protein levels in yeast. PLoS Genet. 11, e1005206 25950722 10.1371/journal.pgen.1005206 PMC4423881

[46] Schreiber A., Stengel F., Zhang Z., Enchev R. I., Kong E. H., Morris E. P., Robinson C. V., da Fonseca P. C., and Barford D. (2011) Structural basis for the subunit assembly of the anaphase-promoting complex. Nature 470, 227–232 21307936 10.1038/nature09756

[47] Heinrich R., Neel B. G., and Rapoport T. A. (2002) Mathematical models of protein kinase signal transduction. Mol. Cell 9, 957–970 12049733 10.1016/s1097-2765(02)00528-2

[48] Johnson H., Eyers C. E., Eyers P. A., Beynon R. J., and Gaskell S. J. (2009) Rigorous determination of the stoichiometry of protein phosphorylation using mass spectrometry. J. Am. Soc. Mass Spectrom. 20, 2211–2220 19783156 10.1016/j.jasms.2009.08.009

[49] Kanshin E., Wang S., Ashmarina L., Fedjaev M., Nifant'ev I., Mitchell G. A., and Pshezhetsky A. V. (2009) The stoichiometry of protein phosphorylation in adipocyte lipid droplets: analysis by N-terminal isotope tagging and enzymatic dephosphorylation. Proteomics 9, 5067–5077 19921680 10.1002/pmic.200800861

[50] Mou S., Sun L., and Dovichi N. J. (2013) Accurate determination of peptide phosphorylation stoichiometry via automated diagonal capillary electrophoresis coupled with mass spectrometry: proof of principle. Anal. Chem. 85, 10692–10696 24144020 10.1021/ac402858a PMC3873722

[51] Gygi S. P., Rochon Y., Franza B. R., and Aebersold R. (1999) Correlation between Protein and mRNA Abundance in Yeast. Mol. Cell. Biol. 19, 1720–1730 10022859 10.1128/mcb.19.3.1720 PMC83965

[52] Lee M. V., Topper S. E., Hubler S. L., Hose J., Wenger C. D., Coon J. J., and Gasch A. P. (2011) A dynamic model of proteome changes reveals new roles for transcript alteration in yeast. Mol. Systems Biol. 7, 514 10.1038/msb.2011.48 PMC3159980 21772262

[53] Zenklusen D., Larson D. R., and Singer R. H. (2008) Single-RNA counting reveals alternative modes of gene expression in yeast. Nat. Struct. Mol. Biol. 15, 1263–1271 19011635 10.1038/nsmb.1514 PMC3154325

[54] Vogel C., Abreu Rde S., Ko D., Le S. Y., Shapiro B. A., Burns S. C., Sandhu D., Boutz D. R., Marcotte E. M., and Penalva L. O. (2010) Sequence signatures and mRNA concentration can explain two-thirds of protein abundance variation in a human cell line. Mol. Syst. Biol. 6, 400 20739923 10.1038/msb.2010.59 PMC2947365

[55] Li J. J., Bickel P. J., and Biggin M. D. (2014) System wide analyses have underestimated protein abundances and the importance of transcription in mammals. PeerJ 2, e270 24688849 10.7717/peerj.270 PMC3940484

[56] Futcher B., Latter G. I., Monardo P., McLaughlin C. S., and Garrels J. I. (1999) A Sampling of the Yeast Proteome. Mol. Cell. Biol. 19, 7357–7368 10523624 10.1128/mcb.19.11.7357 PMC84729

[57] Canelas A. B., Harrison N., Fazio A., Zhang J., Pitkanen J. P., van den Brink J., Bakker B. M., Bogner L., Bouwman J., Castrillo J. I., Cankorur A., Chumnanpuen P., Daran-Lapujade P., Dikicioglu D., van Eunen K., Ewald J. C., Heijnen J. J., Kirdar B., Mattila I., Mensonides F. I., Niebel A., Penttila M., Pronk J. T., Reuss M., Salusjarvi L., Sauer U., Sherman D., Siemann-Herzberg M., Westerhoff H., de Winde J., Petranovic D., Oliver S. G., Workman C. T., Zamboni N., and Nielsen J. (2010) Integrated multilaboratory systems biology reveals differences in protein metabolism between two reference yeast strains. Nature Commun. 1, 145 21266995 10.1038/ncomms1150

[58] Finn B., Harvey L. M., and McNeil B. (2010) The Effect of Dilution Rate upon Protein Content and Cellular Amino Acid Profiles in Chemostat Cultures of Saccharomyces Cerevisiae CABI 039916. Int. J. Food Eng. 6

[59] Gerashchenko M. V., Lobanov A. V., and Gladyshev V. N. (2012) Genome-wide ribosome profiling reveals complex translational regulation in response to oxidative stress. Proc. Natl. Acad. Sci. U. S. A. 109, 17394–17399 23045643 10.1073/pnas.1120799109 PMC3491468

[60] Subtelny A. O., Eichhorn S. W., Chen G. R., Sive H., and Bartel D. P. (2014) Poly(A)-tail profiling reveals an embryonic switch in translational control. Nature 508, 66–71 24476825 10.1038/nature13007 PMC4086860

[61] Li S., Labaj P. P., Zumbo P., Sykacek P., Shi W., Shi L., Phan J., Wu P. Y., Wang M., Wang C., Thierry-Mieg D., Thierry-Mieg J., Kreil D. P., and Mason C. E. (2014) Detecting and correcting systematic variation in large-scale RNA sequencing data. Nat. Biotechnol. 32, 888–895 25150837 10.1038/nbt.3000 PMC4160374

[62] Mehdi A. M., Patrick R., Bailey T. L., and Bodén M. (2014) Predicting the Dynamics of Protein Abundance. Mol. Cell. Proteomics 13, 1330–1340 24532840 10.1074/mcp.M113.033076 PMC4014288

[63] Tuller T., Kupiec M., and Ruppin E. (2007) Determinants of Protein Abundance and Translation Efficiency in S. cerevisiae. PLoS Comput. Biol. 3, e248 18159940 10.1371/journal.pcbi.0030248 PMC2230678

[64] Zur H., and Tuller T. (2013) Transcript features alone enable accurate prediction and understanding of gene expression in S. cerevisiae. BMC Bioinformatics 14, S1 24564391 10.1186/1471-2105-14-S15-S1 PMC3852043

[65] Arava Y., Wang Y., Storey J. D., Liu C. L., Brown P. O., and Herschlag D. (2003) Genome-wide analysis of mRNA translation profiles in Saccharomyces cerevisiae. Proc. Natl. Acad. Sci. U. S. A. 100, 3889–3894 12660367 10.1073/pnas.0635171100 PMC153018

[66] Belle A., Tanay A., Bitincka L., Shamir R., and O'Shea E. K. (2006) Quantification of protein half-lives in the budding yeast proteome. Proc. Natl. Acad. Sci. U. S. A. 103, 13004–13009 16916930 10.1073/pnas.0605420103 PMC1550773

[67] Costello J., Castelli L., Rowe W., Kerhsaw C., Talavera D., Muhammad S., Sims P., Grant C., Pavitt G., Hubbard S., and Ashe M. (2015) Global mRNA selection mechanisms for translation initiation. Genome Biol. 16, 10 25650959 10.1186/s13059-014-0559-z PMC4302535

[68] dos Reis M., Savva R., and Wernisch L. (2004) Solving the riddle of codon usage preferences: a test for translational selection. Nucleic Acids Res. 32, 5036–5044 15448185 10.1093/nar/gkh834 PMC521650

[69] Geisberg, Joseph V., Moqtaderi Z., Fan X., Ozsolak F., and Struhl K. (2014) Global Analysis of mRNA Isoform Half-Lives Reveals Stabilizing and Destabilizing Elements in Yeast. Cell 156, 812–824 24529382 10.1016/j.cell.2013.12.026 PMC3939777

[70] Ingolia N. T., Ghaemmaghami S., Newman J. R. S., and Weissman J. S. (2009) Genome-Wide Analysis in Vivo of Translation with Nucleotide Resolution Using Ribosome Profiling. Science 324, 218–223 19213877 10.1126/science.1168978 PMC2746483

[71] Kertesz M., Wan Y., Mazor E., Rinn J. L., Nutter R. C., Chang H. Y., and Segal E. (2010) Genome-wide measurement of RNA secondary structure in yeast. Nature 467, 103–107 20811459 10.1038/nature09322 PMC3847670

[72] Rechsteiner M., and Rogers S. W. (1996) PEST sequences and regulation by proteolysis. Trends Biochem. Sci. 21, 267–271 8755249

