# Supplementary material for: Direct and Absolute Quantification of over 1800 Yeast Proteins via Selected Reaction Monitoring
Source: Mol Cell Proteomics. 2016 Jan 10;15(4):1309–22. doi: 10.1074/mcp.M115.054288 (PMC4824857; doi:10.1074/mcp.M115.054288)
Supplement: Supplemental Data [file supp_15_4_1309__index.html]

Direct and Absolute Quantification of over 1800 Yeast Proteins via Selected Reaction Monitoring — Direct and Absolute Quantification of over 1800 Yeast Proteins via Selected Reaction Monitoring — Absolute Quantification of the Yeast Proteome — Supplemental Data 

# Direct and Absolute Quantification of over 1800 Yeast Proteins via Selected Reaction Monitoring

## Supplemental Data

- Supplemental methods, figs, tables (.pdf, 11.1 MB) - Supplemental Material including methods, figures
- Supplemental Table 1 (.pdf, 92 KB) - Supplemental Table 1
- Supplemental Table 2 (.pdf, 128 KB) - Supplemental Table 2 + supplemental references
- Supplemental Data 1 (.xlsx, 1.2 MB) - Excel file containing quantification data for all proteins, peptides and QconCAT information
- Supplemental data 3 (.txt, 66 KB) - Fasta file containing QconCAT amino acid sequences
