# Supplementary material for: Direct and Absolute Quantification of over 1800 Yeast Proteins via Selected Reaction Monitoring
Source: Mol Cell Proteomics. 2016 Jan 10;15(4):1309–22. doi: 10.1074/mcp.M115.054288 (PMC4824857; doi:10.1074/mcp.M115.054288)
Supplement: Supplemental Data [file 10.1074_M115.054288_mcp.M115.054288-1.pdf]

Supplementary Material, Methods and Figures

Direct and Absolute Quantification of over 1800 Yeast Proteins via Selected Reaction Monitoring

Craig Lawless*[1], Stephen W. Holman*[2], Philip Brownridge*[2], Karin Lanthaler[1], Victoria M. Harman[2], Rachel Watkins[1], Dean E. Hammond[2], Rebecca L. Miller[2], Paul F. G. Sims[1], Christopher M. Grant**[1], Claire E. Eyers**[2], Robert J. Beynon**[2], Simon J. Hubbard**[1]

[1] Faculty of Life Sciences, University of Manchester, Manchester, UK
[2] Centre for Proteome Research, Institute of Integrative Biology, University of Liverpool, Liverpool, UK

Keywords: absolute quantification; selected reaction monitoring; SRM; stable-isotope labelling; yeast; tandem quadrupole; QconCAT

Contents

Supplementary Material 2
SID-SRM determination of APC subunits 2

Supplementary Methods 3
Protein extraction 3
QconCAT validation and transition 3
Informatic comparison of Q-peptide and endogenous peptide signal response 4
Label-free absolute quantification 5
Analysis of peptide features in sibling pairs 7
Linear regression model building 8

Supplementary Figures 11
Supplementary Figure S1. 13
Supplementary Figure S2. 14
Supplementary Figure S3. 15
Supplementary Figure S4 16
Supplementary Figure S5. 17
Supplementary Figure S6 18
Supplementary Figure S7 19
Supplementary Figure S8. 20
Supplementary Figure S9. 21
Supplementary Figure S10. 22
Supplementary Figure S11. 23
Supplementary Table S1. Anaphase Promoting Complex /Cyclosome quantification by various methods 24
Supplementary Table S2. Translation associated features considered in the multivariate linear regression modelling 25

Supplementary Material

SID-SRM determination of APC subunits

The complete list of APC/C core and regulatory subunit abundance estimates from our study and other proteomic studies is given in Table S1. In addition to abundance estimates for Apc1 and Cdc23, we were also able to estimate an upper limit for 6 core subunits including that of Apc9 at 130 cpc. Apc9 is believed to be present at two copies per complex given its role in stabilisation of the two molecules of Cdc27 found in each APC/C (1). Our quantitative data suggests sub-stoichiometric association of Apc9 in the complex. This raises the intriguing possibility that Apc9 may be the limiting factor in the formation of active APC/C complexes in vivo, where it is known to be essential for APC/C stability and catalytic E3 ligase activity (2); such a scenario would suggest that there are less than 130 functional APC/C complexes per cell. An alternative, but less likely possibility based on current evidence (3), is that Apc9 might function to promote the formation of the more active APC/C dimer recently described (4).

We also determined abundances for the two APC/C regulators Cdc20 and Cdh1, at <130 cpc and 998 ± 156 cpc respectively. The marked differences determined here in the relative amounts of these two co-activator proteins can be explained by a knowledge of their stability and regulation; although Cdh1 levels remain stable throughout the cell cycle, Cdc20 levels are known to fluctuate, consistent with the observation that our measurements derive from an asynchronous cell population (5-7). Our data highlights the diversity of estimated stoichiometry for this complex defined by absolute quantitative proteomics methods (Table S1).

Supplementary Methods

Protein extraction

Accuracy in protein quantification not only relies on the analytical methodology and data analysis pipeline employed, but also, critically, the strategy for protein extraction and sample handling. To minimise potential errors arising from protein loss associated with manipulation of the biological sample, the chemostat grown yeast were lysed following an optimised protocol (8, 9) and the total non-clarified extract used for subsequent protein quantification. No particulate material was removed prior to proteolysis, and protein loss (reproducible or otherwise) was minimised.

QconCAT validation and transition

Each tryptic digest was analysed by LC-MS using a nanoAcquity UPLC™ system (Waters Ltd., Elstree, UK) coupled to a Synapt™ G2 quadrupole-time-of-flight mass spectrometer (Waters Ltd., Elstree, UK) to verify completeness of digestion and to quantify the quantity of QconCAT present in the sample. One µL of the digest (corresponding to approximately the protein equivalent of 100,000 cells) was loaded onto a Symmetry C18 trapping column (5 µm packing material, 180 µm x 20 mm) (Waters Ltd., Elstree, UK) using partial loop injection for 3 min in 0.1% formic acid, 0.1 % acetonitrile at 5 µL min^{-1}. The sample was then resolved on a HSS T3 nanoAcquity C18 analytical column (1.8 µm packing material, 75 µm x 150 mm) (Waters Ltd., Elstree, UK) using a gradient of 97 % A (0.1 % formic acid): 3 % B (0.1 % formic acid in acetonitrile) to 60 % A:40 % B over 60 min at a flow rate of 300 nL min^{-1}. The column was then washed by increasing the percentage of B to 95 % over 2 min and holding at 95 % B for 2.5 min. The column was then re-equilibrated to starting conditions. The column oven temperature was 35 ^{o}C and the autosampler temperature was 7 ^{o}C. A lock mass solution of 500 fmol µL^{-1} of glu-fibrinopeptide B in 0.1 % formic acid in water:acetonitrile [50:50] was infused into the nano-electrospray ionisation (ESI) source from an auxiliary pump at a flow rate of 300 nL min^{-1}. All solvents were LC-MS grade.

The column effluent was introduced into a nano-ESI source operated in positive polarity and fitted with a PicoTip emitter (New Objective, Woburn, MA, USA). The mass spectrometer was calibrated immediately prior to sample analysis using the product ion spectrum of glu-fibrinopeptide B (500 fmol μL^{-1} of glu-fibrinopeptide B in 0.1 % formic acid in water:acetonitrile [50:50]) and operated using a data independent (MS^E) acquisition program with the instrument in V mode. A 1 sec 'low energy' survey scan was performed between *m/z* 50-2000 and a trap cell collision energy of 6 eV. The 'elevated energy' product ion scan was acquired using the same conditions except that the trap cell collision energy was ramped between 15 and 40 eV over the course of the acquisition. The transfer cell collision energy was 4 eV for both scans, and the lock mass was recorded every 30 sec. The data was processed and database searched using ProteinLynx Global Server v2.5.2 (Waters Ltd., Elstree, UK). The data was processed using the following settings; low energy threshold, 100; elevated energy threshold, 20; intensity threshold, 750; lock mass, *m/z* 785.8426. The processed spectra were searched against in-house generated database containing the amino acid sequences of all the QconCATs used in the study. The following settings were applied; automatic settings for precursor and product ion mass tolerance; minimum fragment ion matches per peptide, 8; minimum fragment ion matches per protein, 15; minimum peptide matches per protein, 1; fixed modifications, carbamidomethyl Cys, $^{13}C_6$ Arg and Lys; variable modifications, oxidised Met; number of missed cleavages, 1; false positive rate, 1 %.

Informatic comparison of Q-peptide and endogenous peptide signal response

Our QconCAT design process involved several steps to minimise the likelihood of confounding issues that could degrade the signal from both Q-peptides and the selected endogenous peptides in the yeast proteins. Specifically, we selected surrogate peptides that were not known to be post-translationally modified and used a prediction too, McPred (10), to prioritise peptides with good cleavage contexts to avoid partial cleavage. In addition, our digestion protocol described here and in the Methods was tested and evaluated over several months to optimise conditions for complete digestion. Similarly, as noted above, we routinely examined every QconCAT digest on the Synapt instrument for completeness.

In addition to these steps, we also compared the SRM XIC values from 532 matched pairs of Class A Q-peptides for concordance. The reasoning behind this is illustrated in Supplementary Figure S11 panel A where we expect to observe equal response relative rates from the two yeast peptide SRM values compared to their Q-peptide equivalents, where the pair are from the same yeast protein. This is indeed what we observe in Fig S11B with ~70% of data points lying within a 2-fold difference. The figure also indicates the expected changes when signal is lost from a given SRM value that could be caused by incomplete digestion, sub-stoichiometric post-translational modifications, or chromatographic inconsistencies and poor peak selection. In the cases where large deviations from expectation are observed (red dots, labelled Single) we always selected the largest of the two peptide SRM median values for protein quantification, reasoning that loss of signal from a target peptide is considerably more likely than for QconCAT peptides since these were independently validated and checked.

Label-free absolute quantification

The dataset called "Q-Exactive" was based on a single one dimensional reversed phase separation of the previously described yeast digest using an Ultimate 3000 RSLC™ nano system (Thermo Scientific, Hemel Hempstead, UK) coupled to a Q-Exactive™ mass spectrometer (Thermo Scientific, Hemel Hempstead, UK). The sample (5 µL corresponding to 400,000 cells and 50 fmol glycogen phosphorylase [Uniprot P00489, Waters Ltd., Elstree, UK]) was loaded onto the trapping column (PepMap100, C18, 75 µm X 20 mm) (Thermo Scientific, Hemel Hempstead, UK), using partial loop injection, for 7 min at a flow rate of 4 µL/min with 0.1% (v/v) formic acid. The sample was resolved on the analytical column (Easy-Spray C18 75 µm x 500 mm 2 µm column) (Thermo Scientific, Hemel Hempstead, UK) using a gradient of 96.2% A (0.1% formic acid) 3.8% B (0.1 % formic acid, 80 % acetonitrile) to 50% A 50% B over 240 min at a flow rate of 300 nL min^{-1}. The data-dependent program used for data acquisition consisted of a 70,000 resolution (FWHM at m/z 200) full-scan MS scan (AGC set to 1e6 ions with a maximum fill time of 250 ms) from which the 10 most abundant peaks were selected for MS/MS using a 17,500 resolution scan (FWHM at m/z 200) (AGC set to 5e4 ions with a maximum fill time of 250 ms) with an ion selection window of 3 m/z and a normalised collision energy

of 30. To avoid repeated selection of peptides for MS/MS the program used a 30 sec dynamic exclusion window. The data was processed with Progenesis (v4.1 Nonlinear Dynamics, Newcastle upon Tyne, UK). Samples were aligned according to retention time using a combination of manual and automatic alignment. Default peak picking parameters were applied and features with charges from 1+ to 4+ featuring three or more isotope peaks were retained. Database searching was performed using MASCOT (Matrix Science). A Mascot Generic File was created by Progenesis and searched against the reference proteome set of *S. cerevisiae* from UniProt (6560 proteins) with the sequence of rabbit glycogen phosphorylase B (UniProt accession P00489) added. A fixed carbamidomethyl modification for cysteine and variable oxidation modification for methionine were specified. A precursor mass tolerance of 10 ppm and a fragment ion mass tolerance of 20 mmu were applied. At a *p* value of 0.05, the built in MASCOT decoy search reported a peptide FDR of 0.5%. Label-free absolute quantification was performed following the "Top3" methodology described by Silva et al.(11).

The "SAX" dataset used the on-tip pre-fractionation approach described by Wisniewski and co-workers (12) to increase proteome coverage. Initially 130 µg of whole cell lysate yeast preparation were brought to 250 µL in a solution containing 4% SDS, 100mM Tris/HCl pH 7.6, 0.1M DTT and incubated at 95°C for 5 min. Any DNA in the sample was then sheared by sonication (3 x 10 s pulses) to reduce the sample viscosity and the lysate then clarified by centrifugation at 17,136 x g for 5 min. The sample volume was brought to 400 µL with 100 mM Tris/HCl pH 7.6, the sample cooled on ice, and 100 µL of 100% TCA added to precipitate protein. Precipitation was carried out overnight on ice after which the precipitate was washed twice with ice-cold acetone, dried (though not fully), re-suspended in a solution containing 8 M urea, 0.1 M Tris-HCl (pH 8.5) and 0.1 M DTT, and the protein reduced by incubation for 10 min at 60 °C. Alkylation was then performed by adding iodoacetamide in 0.1 M Tris-HCl (pH 8.5) to a final concentration of 50 mM and incubation of the sample for 30 min in the dark at room temperature. Further 0.1 M Tris-HCl (pH 8.5) was added, to dilute the urea to 6 M, after which endoproteinase LysC was added at 1:50 enzyme:protein ratio, and the mixture incubated for 4 h at 37 ^{o}C. Following this, the urea concentration was further diluted

down to 1.5 M with 25 mM ammonium bicarbonate before adding trypsin (1:50 enzyme:protein ratio) for an overnight incubation at 37 °C. The peptide digest was desalted using a Harvard apparatus Macro spin column, and eluted from the C18 matrix using 70% acetonitrile, dried by vacuum centrifugation prior to preparation for anion exchange-based peptide fractionation as described previously (12). Briefly, peptides were separated on a pipette-based anion exchanger 'column', assembled by stacking 10 layers of a 3 M Empore Anion Exchange disk (consisting a polystyrenedivinylbenzene copolymer, modified with quaternary ammonium groups) into a 200 µL micropipette tip. Peptides were initially dissolved and loaded on the column, in 200 µL of Britton and Robinson buffer composed of 20 mM CH_3COOH, 20 mM H_3PO_4, 20 mM H_3BO_3, and NaOH, at pH 11. For elution of fractions, Britton and Robinson buffer solutions titrated to pH 8, 6, 5, 4 and 2 using NaOH were used. Eluted peptides were also desalted using C18 StageTip plugs as described by Rappsilber and co-workers (13). Each elution was then dried by vacuum centrifugation, re-suspended in 0.05% TFA prior to LC-MS/MS. LC-MS/MS was performed using an Ultimate 3000 RSLC™ nano system (Thermo Scientific, Hemel Hempstead, UK) coupled to a Q-Exactive™ mass spectrometer (Thermo Scientific, Hemel Hempstead, UK) using the previously described method. The SAX dataset was processed with MaxQuant (v. 1.3.0.5, (14) using the Andromeda search engine(15). The sequence database was the reference proteome set of *S. cerevisiae* from UniProt (6560 proteins). A fixed carbamidomethyl modification for cysteine and variable oxidation modification for methionine were specified. A precursor mass tolerance of 10 ppm and a fragment ion mass tolerance of 20 mmu were applied and a 1% FDR threshold at both peptide and protein level. All other parameters were left at default settings. Label-free absolute quantification was performed following the "Top3" methodology described by Silva and co-workers (11) but instead of using an internal standard for calibration, the total content of a yeast cell was assumed to be 60 million molecules.

Analysis of peptide features in sibling pairs

We examined the peptides in sibling pairs from the same parent protein, where both had been classified as Type A, for enrichment in selected features which could explain systematic differences in the log2 ratio of X/Y abundances. In all cases, the

peptides were denoted X and Y so that the X value was always the greater of the tow. We noted a statistically significant enrichment in certain features of the X and Y peptides in pairs with $\log_2$ ratios above and below the median, most notably increased missed cleavage potential in the native protein context of the lower abundance Y peptides (10), as shown in Supplementary Figure 3. Other features considered amino acid content and predicted post-translational modifications. As noted the most prominent feature that explains the data is an enrichment for missed cleavage potential in the native protein context of the lower abundance Y peptides according to our predictor MC:Pred (10), as well as incidence of dibasic sites (16), and the presence of tryptophan in the X peptide. We are aware of no precedent for this latter observation but observed a modestly significant distinction between X/Y ratios conditioned on this feature (Supplementary Figure 3). However, we elected not to use this directly when selecting peptides for protein level quantification due to the borderline significance and no clear experimental rationale.

Linear regression model building

Several experimental parameters are associated with translation rates in yeast including ribosomal density of translating transcripts measured by sucrose gradient sedimentation (17), ribosomal footprint profiling by RNA-seq (18), propensity for mRNA folding at the nucleotide level (19), mRNA poly-adenine tail length (20), codon adaptation of specific transcripts (translation adaptation index, tAI) (21), and RNA-binding protein immunoprecipitation (RIP) enrichment by key translation factors (22). Other characteristics that influence final protein expression levels are 5′ and 3′ UTR lengths (23, 24), transcript half-life (25) and protein half-life (26), as well as sequence recognition motifs involved in protein degradation, such as PEST sequences (27). We adopted an iterative, sequential approach to the multivariate linear regression modeling using features listed in Supplementary Table 3. The linear model was constructed by sequentially adding translation-related metrics to the model in descending order based on their observed correlation with protein abundance alone. Features were retained if they significantly contributed to the regression model. Due to the under-sampled nature of most large-scale biological studies, missing values are quite common, and therefore the intersection of data

between orthogonal studies resulted in a decrease in data points to model at progressive stages.

The final model derived is shown below in Equation 1. This includes additional features, not yet referred to; enriched transcript binding by translation factors (*CAF20 IP*), ribosomal occupancy (*Ingolia TE*)(18), transcript half-life (*mRNA HL*)(25), and an interaction term involving mRNA abundance, median poly-A tail length (*polyA TL*)(20), 5′UTR length and 3′UTR length. We believe this is one of the most accurate models of global protein translation in yeast to date, predicting protein abundance (PA, below) from matched transcript abundance (the *mRNA* term below) in combination with the other terms. As discussed in the main paper however, although we observe a modest increase in the model's ability to predict protein abundance from transcript abundance using post-transcriptional/translational features, most of the variance is explained by the transcriptome data.

Equation 1:

$$\begin{aligned}\mathbf{log_{10}}(\boldsymbol{PA}) = {} & \mathbf{4.101} \\ & + \mathbf{0.5281} \\ & \times \mathbf{log_{10}}(\boldsymbol{mRNA}) + \mathbf{3.009} \times \boldsymbol{tAI} + \mathbf{0.2648} \times \boldsymbol{CAF}\mathbf{20}\ \boldsymbol{IP\ Enrichment} \\ & + \mathbf{0.2581} \times \boldsymbol{Ingolia\ TE} + \mathbf{0.2562} \times \mathbf{log_{10}}(\boldsymbol{mRNA\ HL}) + \mathbf{1.08}\boldsymbol{E}^{-\mathbf{7}} \\ & \times \mathbf{log_{10}}(\boldsymbol{mRNA}) \colon \mathbf{3`}\boldsymbol{UTR\ Length} \colon \mathbf{5`}\boldsymbol{UTR\ Length} \colon \boldsymbol{polyA\ TL}\end{aligned}$$

We also analysed our transcriptome and proteome data following the Ranged Major Axis (RMA) regression modelling of Csardi and colleagues (28), comparing this to the Ordinary Least Squares (OLS) modelling parameters, using the R-toolbox *lmodel2*. The RMA approach models noise in both the dependent and independent variables (in our case, the proteome and transcriptome respectively) and produces a symmetric model. The best-fit lines are shown in Supplementary Figure S10. The increased slope derived from the RMA modelling is consistent with the Csardi modelling study which considers a variety of datasets, though few with matched transcriptome and proteome from the same yeast. This result suggests that so-called potentiation model of gene expression in yeast is prevalent, where the translational control step follows and amplifies the transcriptional level. We note however, that

even against such a model considerable gene-level variation must exist. This is self-evident from Figure 5B where a huge variation in protein:mRNA ratios are observed. To properly reconcile this data requires an accurate comprehensive measurement of both protein degradation and synthesis rates. Although the inclusion of protein turnover improves the quality of the basic model, considerable variance remains unexplained suggesting better data with better coverage are still needed.

Supplementary Figures

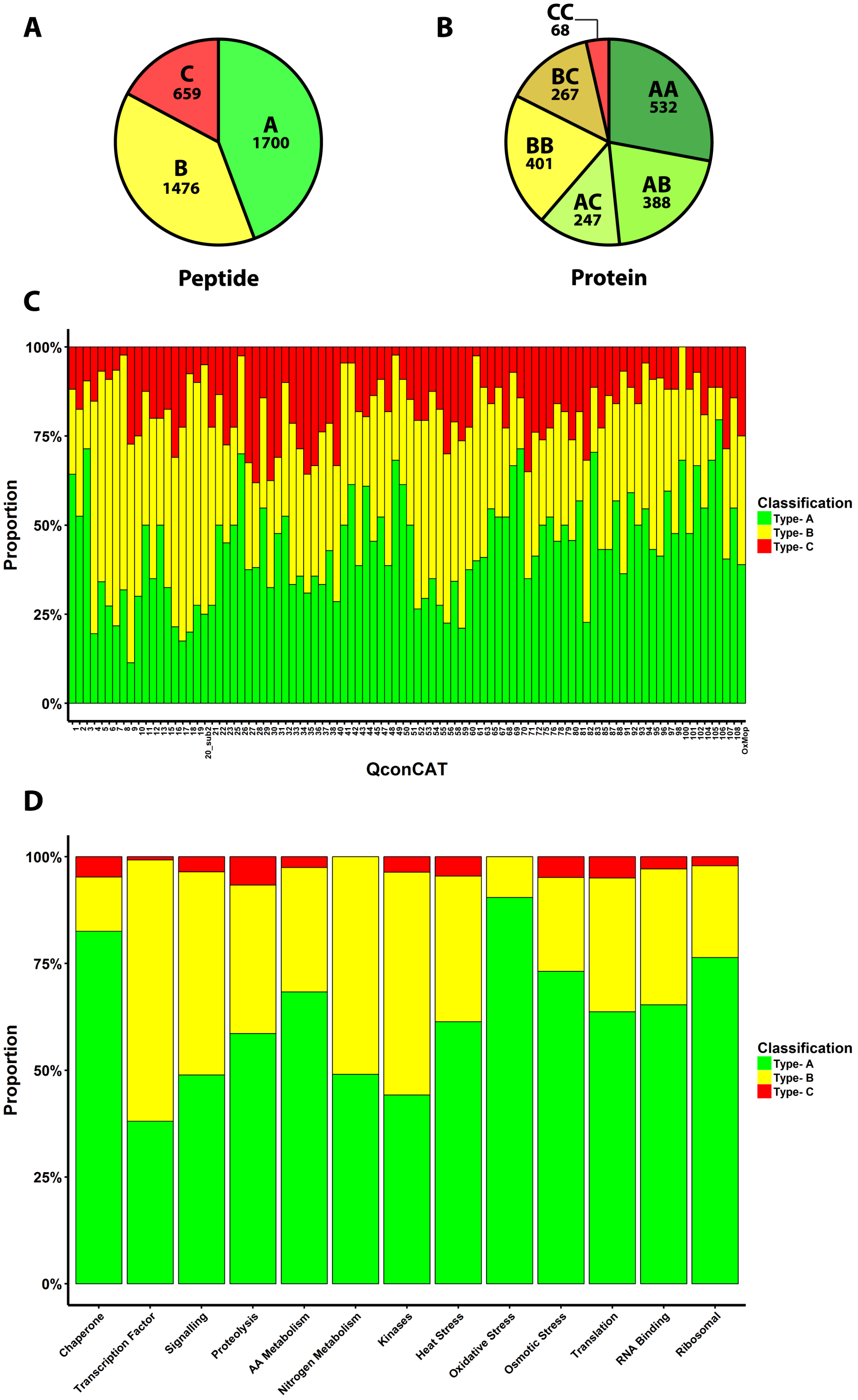
A
C
659
A
1700
B
1476
Peptide
B
CC
68
BC
267
AA
532
BB
401
AC
247
AB
388
Protein
C
100%
75%
50%
25%
0%
Proportion
QconCAT
Classification
Type- A
Type- B
Type- C
D
100%
75%
50%
25%
0%
Proportion
Chaperone
Transcription Factor
Signalling
Proteolysis
AA Metabolism
Nitrogen Metabolism
Kinases
Heat Stress
Oxidative Stress
Osmotic Stress
Translation
RNA Binding
Ribosomal
Classification
Type- A
Type- B
Type- C

Supplementary Figure S1. .

Quantification classifications. Q-peptides are classified as type 'A' (both 'light' native yeast and 'heavy' QconCAT-derived reference peptides observed; green), type 'B' (only 'heavy' QconCAT-derived reference peptides quantifiable; amber) and type 'C' (neither native not reference peptide quantifiable; red). (A): Total number of peptides of each type in the dataset; (B): Number of proteins quantified for each of the combinations of peptide classifications; (C): Proportions of A, B and C type peptide quantifications across all QconCATs reported in this study are shown as stacked bars. (D): As above, but displaying data according to protein functional classes.QconCATs were designed and synthesized by functional groupings. Protein quantification classifications Protein type 'A' (green) includes AA, AB and AC peptide classifications, protein type 'B' (orange) includes BB and BC peptide classifications, protein type 'C' (red) represents the CC peptide group.

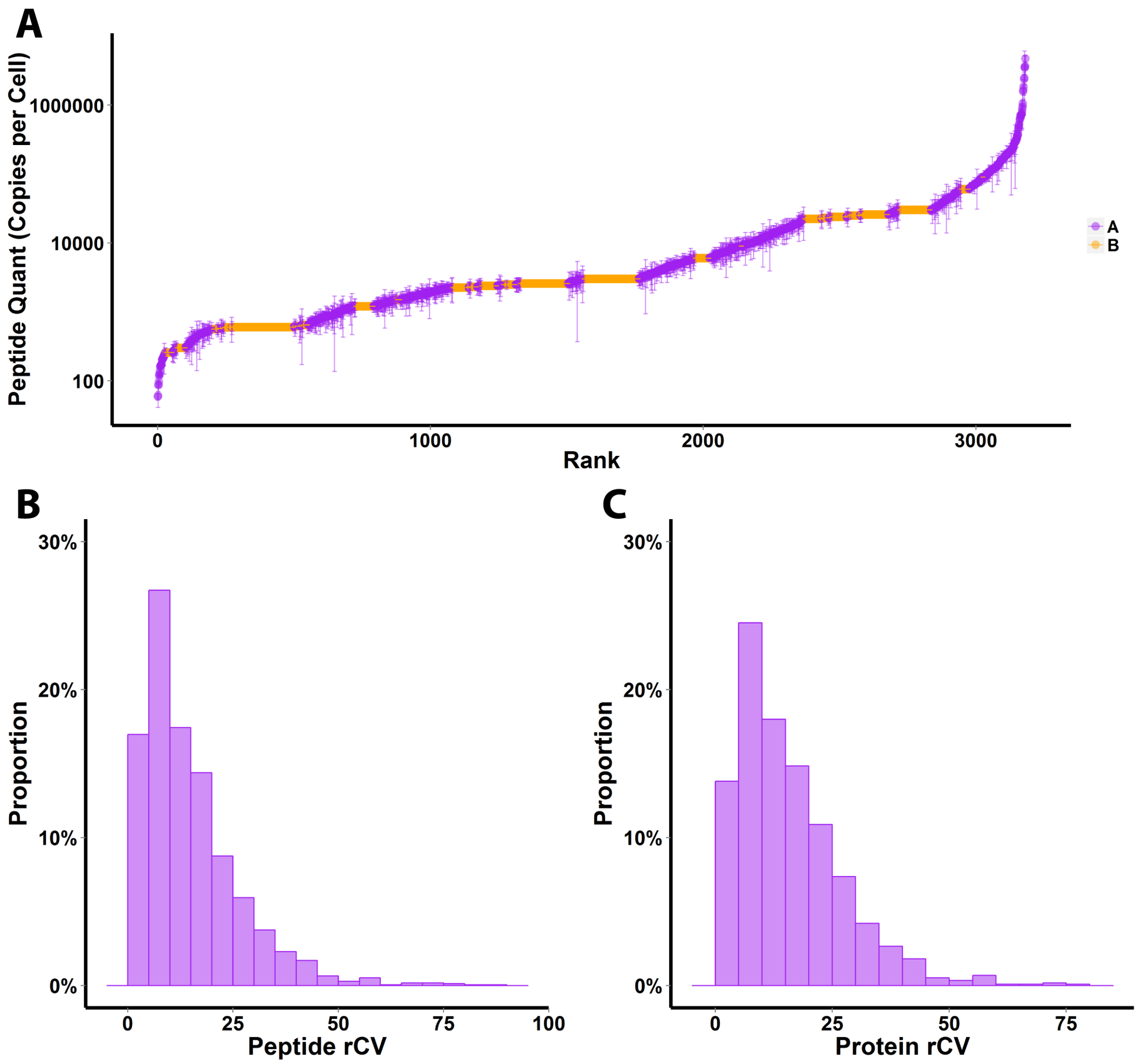

Supplementary Figure S2.

A: An S-curve scatter plot showing peptide quantification values in copies per cell (cpc) ranked in ascending order. The A-type peptides are shown in purple and the upper quantification limit for B-type peptides are shown in orange. The error bars on the A-type peptides show the robust standard deviation (rSD) across the biological replicates. B: The distribution of robust coefficient of variance values (rCVs) are shown in histograms at the peptide level, and in C: at the protein level. The median rCVs are 11.4% and 12.6% respectively, for peptides and proteins.

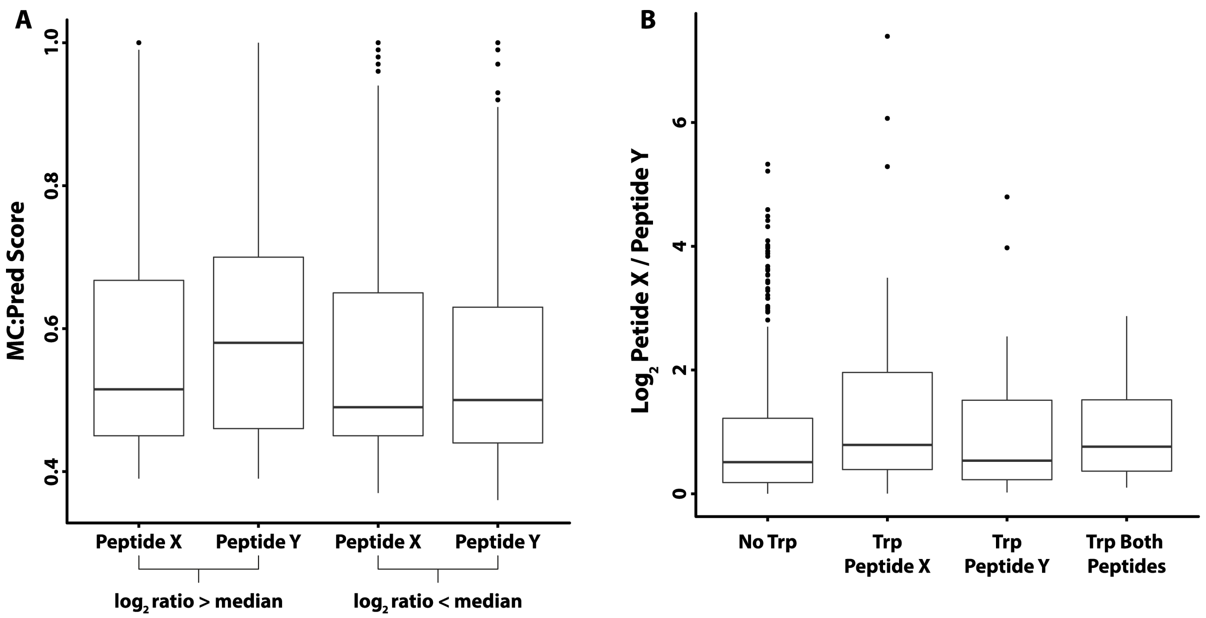

Supplementary Figure S3.

Boxplots showing distinguishing features between sibling peptides for proteins quantified by two A-type peptides. A: The predicted missed-cleavage propensity scores (MC:Pred) are shown for sibling peptides for proteins where sibling A-types agree (log ratio < median log ratio) and those that do not agree (log ratio > median log ratio). Sibling peptides are labelled as either X or Y, so that peptide X quantification > peptide Y in all cases. A significant difference in MC:Pred score is observed for Y peptides in proteins where sibling peptides do not agree *versus* proteins where sibling peptides do agree (Wilcoxon Rank Test, $p<0.001$). B: The log ratio of sibling peptide quantifications (using the same XY nomenclature) is shown for proteins quantified by two peptides. Proteins were classified by the presence of tryptophan in the Q-peptides where either none, one or both peptides contain tryptophan. There is a small but modestly significant difference between log ratio for proteins where peptide X (only) contains a tryptophan and those proteins where neither contain tryptophan (Wilcoxon Rank Test, $p<0.05$). We highlight this test to illustrate the process we carried out to determine if there were any biases in amino acid composition leading to signal loss or disparity. This was the only case, and owing to the modest significance we elected not to use this in the final calculations.

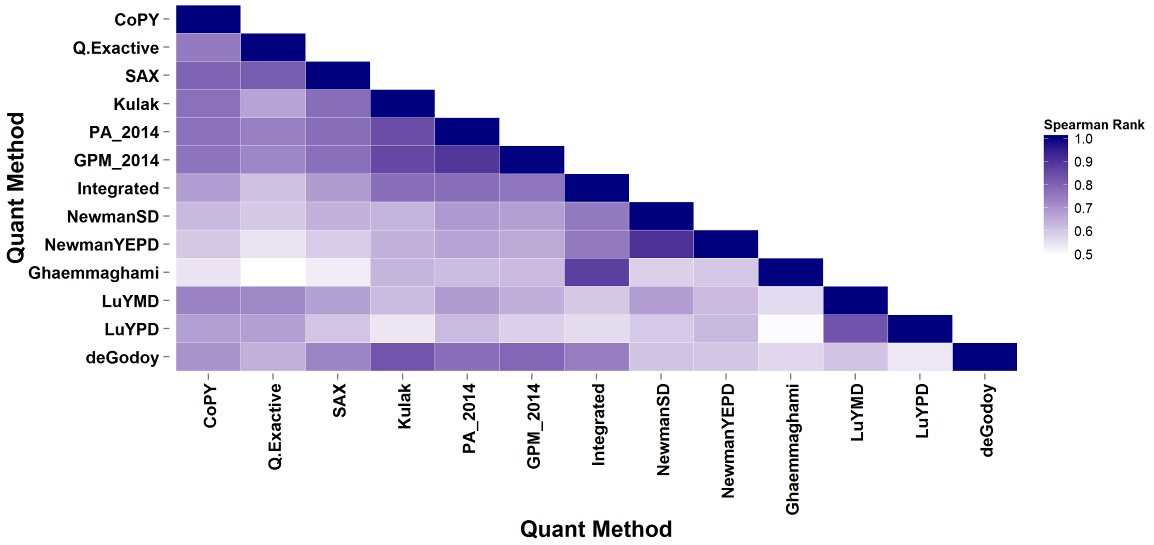

Supplementary Figure S4

Spearman-correlation heatmap showing the comparison of PaxDB yeast proteome, Kulak and COPY datasets for all 1167 proteins quantified in the COPY project. (PA_2014 indicates spectral counting from Peptide Atlas Yeast build 2014 and GPM_2014 indicated spectral counting from all 2014 update of Yeast data in GPM database)

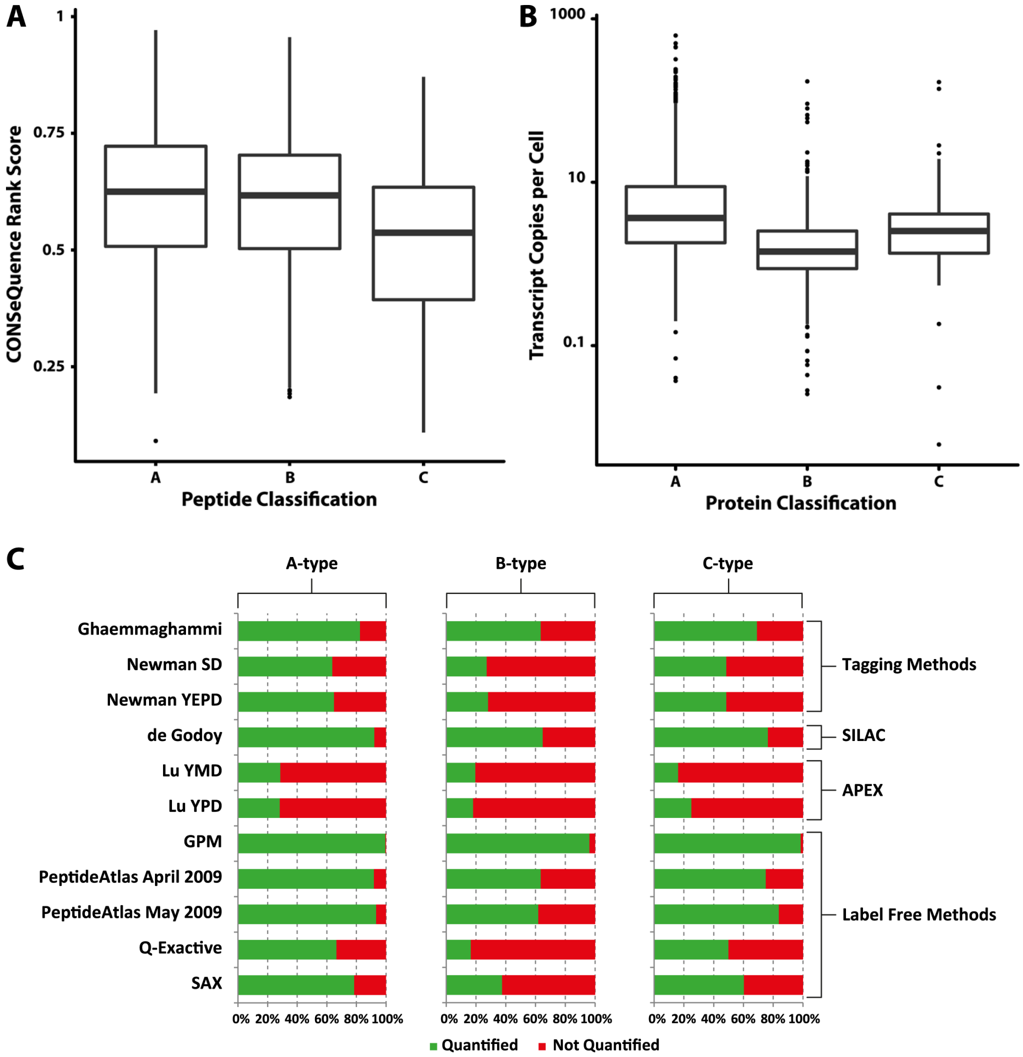

Supplementary Figure S5.

A: Peptide "detectability" prediction scores. The boxplot here shows the predicted detectability score using CONSeQuence of the three observed Q-peptide categories, where the higher score equates to a predicted higher detectability. Peptides were classified by quantification type as before: Type A where reliable data for both the reference and analyte peptide are observed, type B where only data for the reference Q-peptide is available, and type C where we do not see any data for either reference or analyte peptides. Type C peptides are significantly poorly predicted compared to A and B. B: Transcript abundance boxplots for protein quantification categories. Proteins were classified based on their peptide categories. Type A proteins have at least one type A peptide, Type B proteins have no type A but at least one type B, and Type C if neither peptide is type A or B. The boxplots show the respective transcript levels of the three protein categories, plotting transcript copies per cell values as described in the Methods on a log scale. Notably, type B proteins have significantly lower transcript levels compared to type A and C, suggesting they are also likely to be low abundance at the protein level. C: Barcharts showing the proportion of proteins quantified (green) and not quantified (red) across the three protein classifications, broken down for each quantification dataset obtained from PaxDb as well our two label free acquisitions (Q-Exactive and SAX, See Supplementary methods). Of note, there are always proteins that were missed by other methods compared to the successful COPY A-type proteins. Similarly, the relative fraction of COPY B-type quantifications that were unreported by other methods is expanded in comparison to the A-type data, consistent with the fact that they are generally low abundance and hard to quantify by any method.

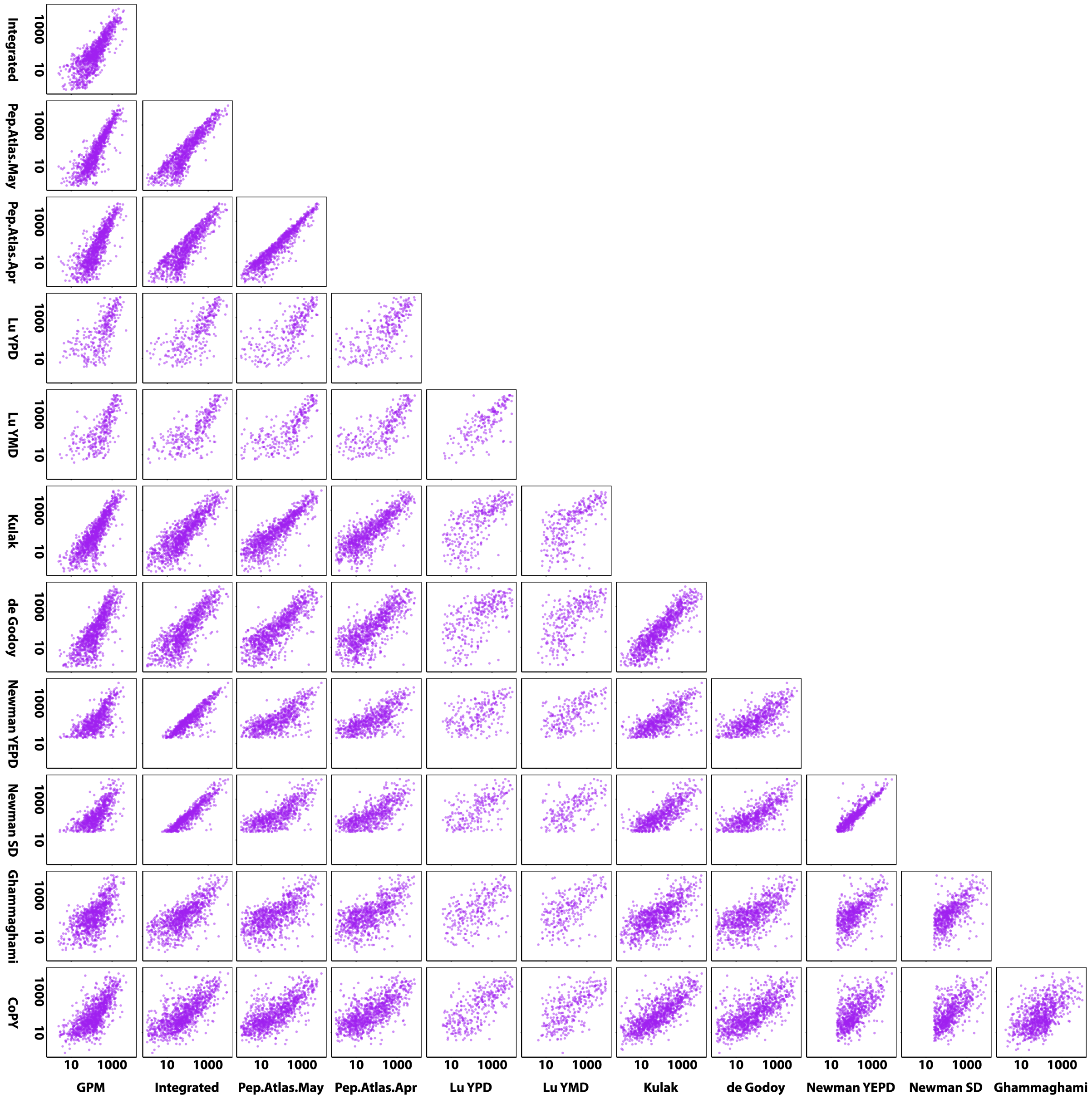

Supplementary Figure S6

Scatter plots of all *versus* all pairwise comparisons of the PaxDb yeast proteome, COPY and Kulak datasets, considering the subset of 1167 proteins quantified by COPY. All values are in parts per million (ppm). COPY quantified proteins were converted to ppm assuming an estimated 108 million protein molecules per cell.

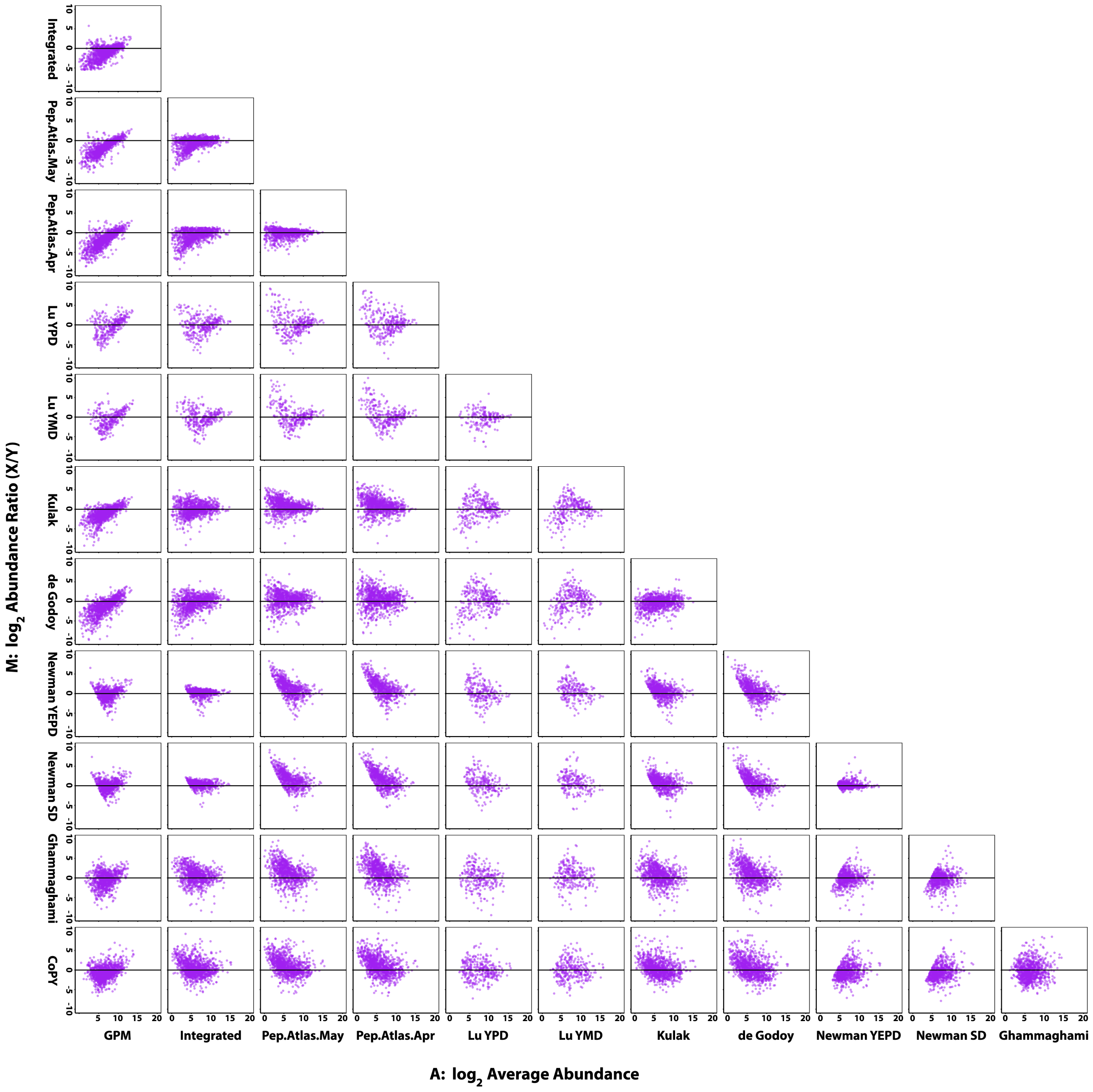

Supplementary Figure S7

M *vs* A-style plots for all *versus* all pairwise comparisons of PaxDb yeast proteome datasets, Kulak and the COPY datasets for all 1167 proteins quantified by COPY. All values are in parts per million (ppm). COPY quantified proteins were converted to ppm assuming the estimated 108 million protein molecules per cell. The x-axis is calculated for each protein common between two datasets as $0.5\times \log_2(Dataset\ A \times Dataset\ B)$ and y-axis calculated as $\log_2(Dataset\ B \div Dataset\ A)$, where Dataset A is labelled on the x-axis and Dataset B is labelled on the y-axis. This representation highlights systematic differences between methodologies which are often apparent at low abundances.

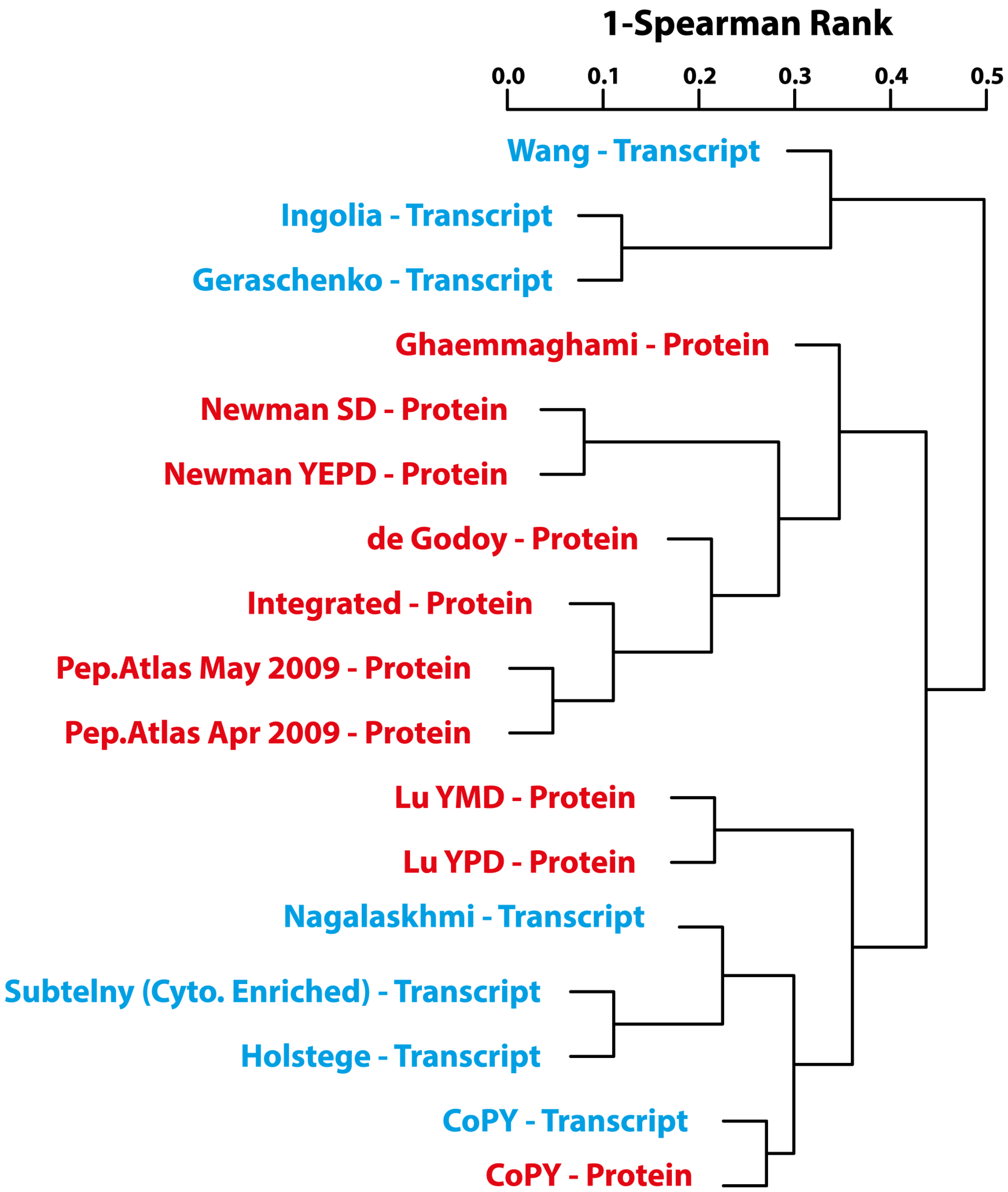

Supplementary Figure S8.

Clustering of independent proteome and transcriptome datasets. The dendrogram is based on the non-parametric comparison of independent proteomes and transcriptomes of yeast. Proteomic datasets (red) were obtained from CoPY project and PaxDb (Wang et al, 2012). The transcriptome datasets (blue) were obtained from the CoPY project, Wang (29) Ingolia (18), Gerashchenko (30), Nagalakshmi (31), Subtelny (20) and Holstege (32).

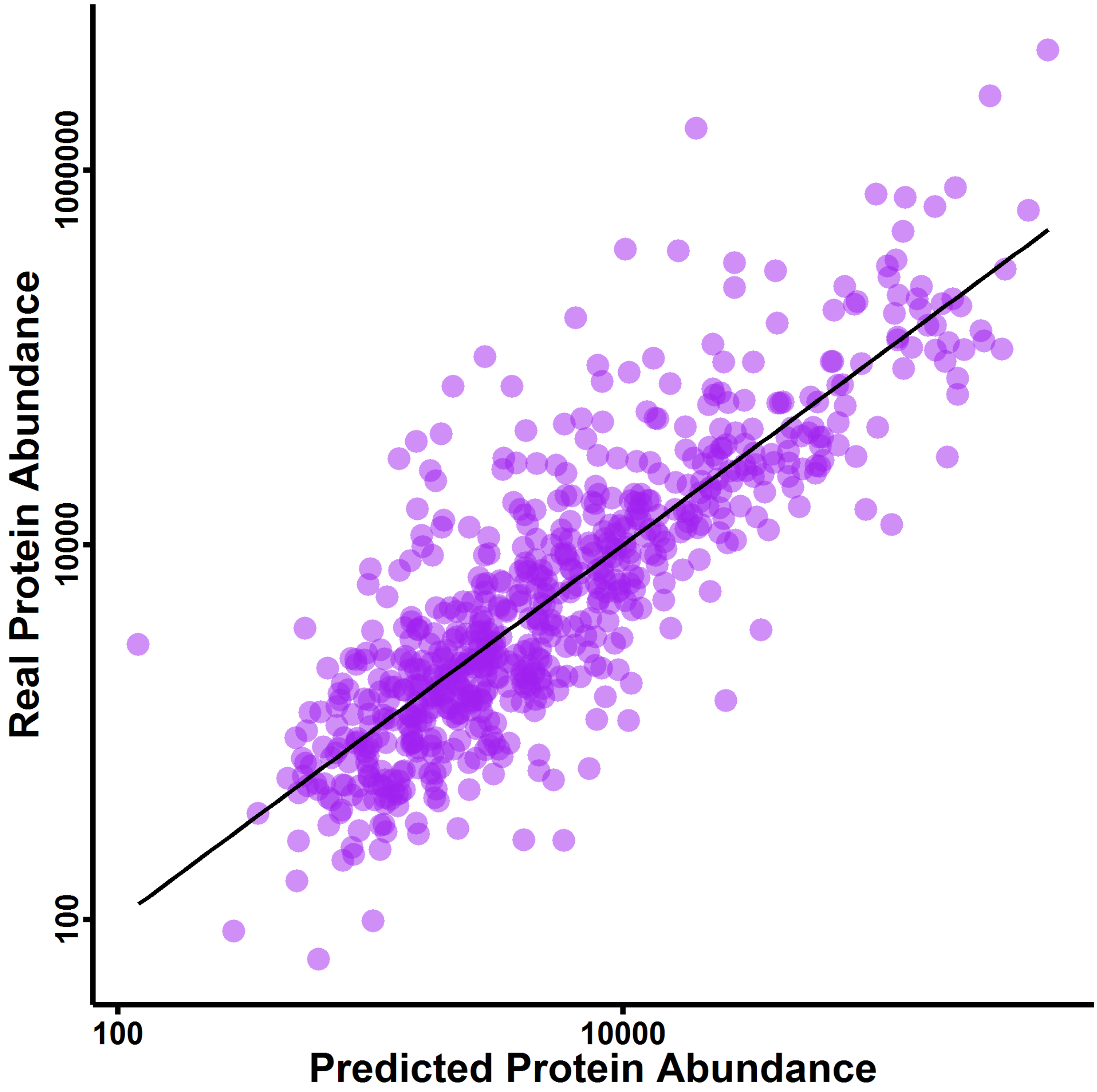

Supplementary Figure S9.

Relationship between actual and predicted protein abundance derived from linear modelling. The predicted and actual protein cpc values are highly correlated for the P1200 protein set under consideration, with a Spearman correlation of 0.84, demonstrating good predictive accuracy. The model uses using 60% of our protein abundance measurements, due to loss of some data points when combining features containing some missing data. As expected, this is common in post-genomic data sets which do not cover all genes/proteins/transcripts.

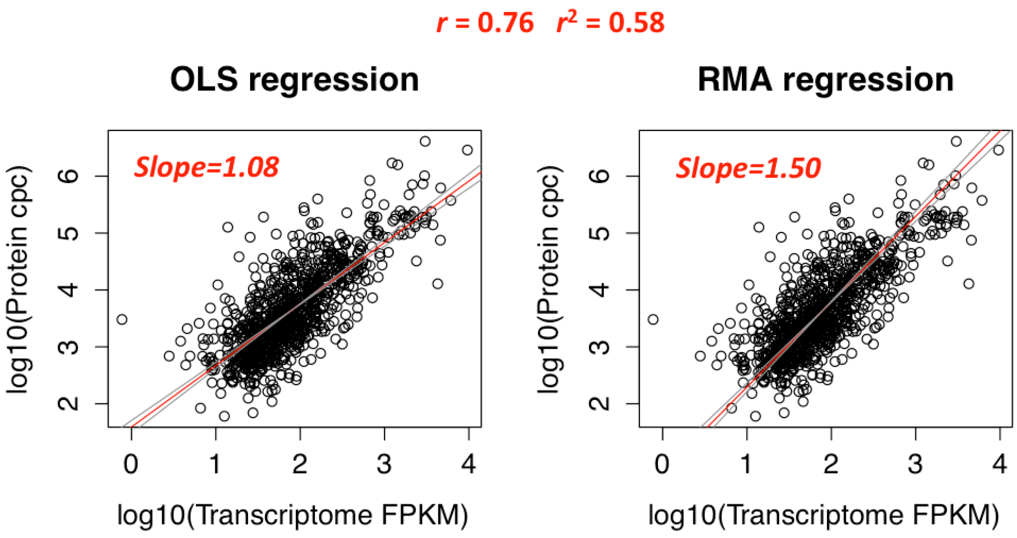

Supplementary Figure S10.

Relationship between protein and transcriptome abundance modeled with the R-package *lmodel2*. Two modelling approaches are shown, which both yield identical correlation coefficients, although they treat noise in the variables differently and hence generated different best-fit lines and slopes. The RMA modelling suggests a non-unitary relationship between protein and RNA abundance.

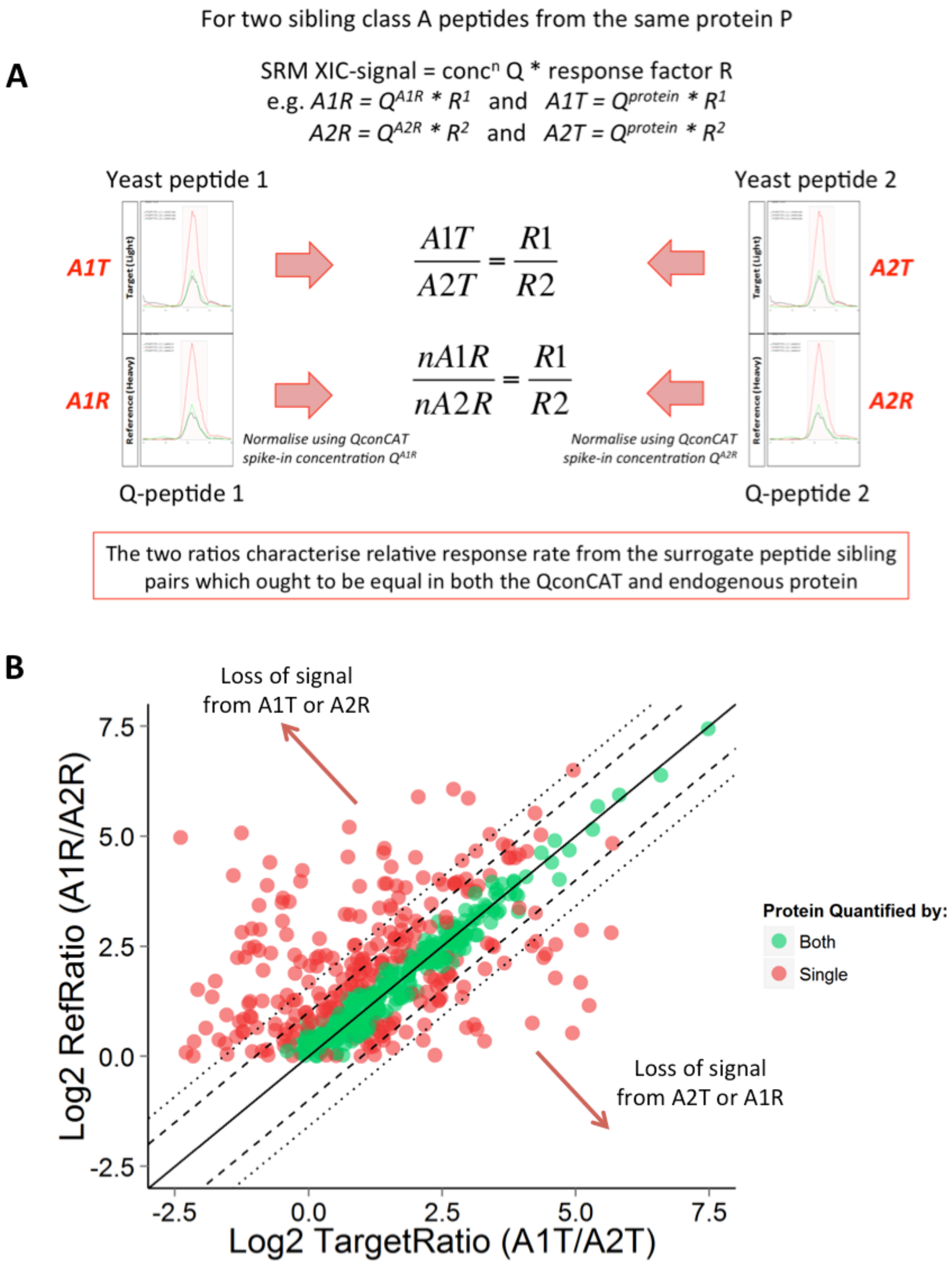

Supplementary Figure S11.

Comparison of SRM values for matched sibling peptides in QconCATs and endogenous yeast peptides. Panel A illustrates how the underlying response factor ratio of individual peptides in a pair should be concordant between the QconCAT and yeast peptides. This presumes that the digestion of the peptides and post-translational modifications are consistent between reference and target, and deviation from this ideal would lead to signal loss from one of the SRM values. Panel B illustrates the actual values calculated for 532 peptide pairs from our dataset plotting values where A1R > A2R in all cases. The majority of points, coloured green, lie close to the solid line representing expectation (x=y) and where final protein quantification is the median value from all bio-replicates. The dashed line represents a two-fold discrepancy between the ratios and the dotted line represents a three-fold discrepancy, both indicating a 2 or 3 fold signal loss in either the target or reference peptides. Around 70% of all values lie with a 2-fold ratio difference.
