# Supplementary material for: Direct and Absolute Quantification of over 1800 Yeast Proteins via Selected Reaction Monitoring
Source: Mol Cell Proteomics. 2016 Jan 10;15(4):1309–22. doi: 10.1074/mcp.M115.054288 (PMC4824857; doi:10.1074/mcp.M115.054288)
Supplement: Supplemental Data [file 10.1074_M115.054288_mcp.M115.054288-2.pdf]

*Supplementary Table S1. Anaphase Promoting Complex /Cyclosome quantification by various methods*

| | Gene Name | Stoichio-metry[a] | CoPY[b] | Kulak | Lu YPD | | Lu YMD | | Newman YEPD | Peptide Atlas May2009 | | GPM | | De Godoy | | Newman SD | PaxDB Integrated | | Ghaemmaghami | |
|---|---|---|---|---|---|---|---|---|---|---|---|---|---|---|---|---|---|---|---|---|
| **Subunit** | | | | cpc | ppm | ratio[c] | ppm | ratio | ppm | ppm | ratio | ppm | ratio | ppm | ratio | ppm | ppm | ratio | ppm | ratio |
| Apc1 | YNL172W | 1 | 260 | 28 | 4.18 | 1 | 17.7 | 1 | NA | 0.7 | 1 | 14.0 | 1 | 5.40 | 1 | NA | 1.4 | 1 | 4.0 | 1 |
| Apc2 (Rsi1) | YLR127C | 1 | ND | 43 | NA | - | 10.1 | 0.6 | NA | NA | - | 103 | 7.3 | NA | - | NA | 2.9 | 2.0 | 9.6 | 2.4 |
| Apc4 | YDR118W | 1 | <500 | 122 | NA | - | 6.5 | 0.3 | NA | NA | - | 132 | 9.5 | 2.63 | 0.5 | NA | 4.0 | 2.8 | 30 | 7.5 |
| Apc5 (Rmc1) | YOR249C | 1 | <480 | 23 | NA | - | NA | - | NA | NA | - | 77.7 | 5.5 | NA | - | NA | 1.9 | 1.3 | 5.8 | 1.5 |
| Apc10 (Doc1) | YGL240W | 1 | <500 | 151 | NA | - | NA | - | NA | 1.1 | 1.6 | 84.5 | 6.0 | 0.43 | 0.1 | NA | 3.5 | 2.4 | 30 | 7.5 |
| Apc11 | YDL008W | 1 | <500 | NA | NA | - | NA | - | 56.3 | NA | - | 22.2 | 1.6 | 0.44 | 0.1 | 57.11 | 24.5 | 17 | 8.4 | 2.1 |
| Cdc23 | YHR166C | 2 | 830 | 72 | NA | - | NA | - | NA | 8.3 | 12 | 140 | 10 | 0.04 | 0.0 | NA | 7.7 | 5.4 | 1.8 | 0.5 |
| Cdc16 | YKL022C | 2 | ND | 63 | NA | - | NA | - | NA | 0.1 | 0.1 | 235 | 17 | 215 | 40 | NA | 11.9 | 8.3 | 61 | 15 |
| Cdc27 (Apc3/Snb1) | YBL084C | 2 | ND | 70 | 56.5 | 14 | 11.5 | 0.6 | 21.4 | 0.1 | 0.2 | 111 | 8 | 0.04 | 0.0 | NA | 9.0 | 6.3 | 13 | 3.3 |
| Apc9 | YLR102C | 2? | <130 | NA | NA | - | NA | - | NA | NA | - | 35.9 | 2.6 | NA | - | NA | 0.8 | 0.6 | NA | - |
| Mnd2 | YIR025W | 1 | ND | 8 | NA | - | NA | - | NA | NA | - | 119 | 8.5 | NA | - | NA | 2.8 | 1.9 | NA | - |
| Apc13 (Swm1) | YDR260C | 1 | ND | NA | NA | - | NA | - | NA | 3.8 | 5.5 | 74.2 | 5.3 | NA | - | NA | 3.8 | 2.6 | NA | - |
| Cdc26 (Hit3/Scd26) | YFR036W | 2 | <4780 | NA | NA | - | NA | - | NA | NA | - | NA | - | NA | - | NA | NA | - | NA | - |
| **Co-activators** | | | | | | | | | | | | | | | | | | | | |
| Cdc20 | YGL116W | ND | <130 | | 8.74 | 2.1 | NA | - | 23.5 | NA | - | 153 | 11 | NA | - | NA | 8.7 | 6.0 | NA | - |
| Cdh1 | YGL003C | ND | 1000 | | NA | - | NA | - | NA | NA | - | 17.6 | 1.3 | NA | - | NA | 0.4 | 0.3 | NA | - |

[a] based on Schreiber et al, 2011. [b]CPC (nearest 10). [c]Ratio w.r.t. Apc1

Quantification of the various anaphase promoting complex/cyclosome (APC/C) protein system components, along with co-activators. The yeast systematic ORF names are provided for the subunits and co-activators, as well as standard gene (SGD) names, along with the putative stoichiometry established from structural studies by Schreiber and colleagues (1). These are compared to known quantification values from various high-throughput studies in yeast, taking the ppm values from PaxDb (33), either derived by PaxDb or by the following studies: Lu YPD/YMD (34), Newman YPD/SD (35), de Godoy (36), Ghaemmaghami (37). Stoichiometric estimates are presented, where possible, expressed as a ratio w.r.t the Apc1 value. For example, we have calculated the stoichiometry of Cdc23, two
