# Supplementary material for: Direct and Absolute Quantification of over 1800 Yeast Proteins via Selected Reaction Monitoring
Source: Mol Cell Proteomics. 2016 Jan 10;15(4):1309–22. doi: 10.1074/mcp.M115.054288 (PMC4824857; doi:10.1074/mcp.M115.054288)
Supplement: Supplemental Data [file 10.1074_M115.054288_mcp.M115.054288-3.pdf]

Supplementary Table S2. Translation associated features considered in t[illegible] multivariate linear regression modelling.

The second and third columns show the regression (R^2) and Spearman Ra[illegible] correlation of each feature to our measured protein abundance.

Feature	R^2 to Protein Abundance	Spearman Ra[illegible] Protein Abu[illegible]
**mRNA abundance†**	0.58***	0.73***
**tAI**	0.53***	0.69***
**CAF20 IP Enrichment**	0.42***	-0.64***
**EAP1 IP Enrichment**	0.42***	-0.63***
**eIF4G2 IP Enrichment**	0.34***	-0.58***
**PARS folding score**	0.31***	0.52***
**eIF4G1 IP Enrichment**	0.27***	-0.52***
**Ribosomal Occupancy**	0.25***	0.51***
**Ingolia Translation efficiency†§**	0.21***	0.49***
**Subtelny Translation efficiency †§**	0.23***	0.52***
**Ribosomal Density**	0.20***	0.49***
**Median Poly-A tail length**	0.19***	-0.43***
**Geraschenko Translation efficiency †§**	0.15***	0.42***
**eIF4E IP Enrichment**	0.14***	0.37***
**mW**	0.06***	-0.24***
**Length of transcript**	0.05***	-0.23***
**Protein Half Life†**	0.04***	0.23 ***
**Transcript Half-Life†**	0.001	-0.08*
**5`UTR Length**	0.001	-0.08*
**Pab1 IP Enrichment**	0	-0.001
**3`UTR Length**	0.002	0.04

†Indicates log transformation for calculation of R^2.
§Translation efficiency calculated as the ratio between ribosomal bound t[illegible] RPKM and all transcript RPKM from RNA-seq analysis.
Asterisks depict P-value significance as follows * P≤0.01, ** P≤0.001, *** P[illegible]

Supplementary Data File 1: Excel file containing peptide and protein level classification and quantification for each bioreplicate. Additionally, RNA-seq FPKM values for our SOLiD sequencing runs are reported, as well as label-free intensity data for two independent quantifications of the yeast proteome (see Methods).

References

1. Schreiber, A., Stengel, F., Zhang, Z., Enchev, R. I., Kong, E. H., Morris, E. P., Robinson, C. V., da Fonseca, P. C., and Barford, D. (2011) Structural basis for the subunit assembly of the anaphase-promoting complex. *Nature* 470, 227-232
2. Passmore, L. A., McCormack, E. A., Au, S. W., Paul, A., Willison, K. R., Harper, J. W., and Barford, D. (2003) Doc1 mediates the activity of the anaphase-promoting complex by contributing to substrate recognition. *The EMBO journal* 22, 786-796
3. Barford, D. (2011) Structure, function and mechanism of the anaphase promoting complex (APC/C). *Quarterly reviews of biophysics* 44, 153-190
4. Passmore, L. A., Booth, C. R., Venien-Bryan, C., Ludtke, S. J., Fioretto, C., Johnson, L. N., Chiu, W., and Barford, D. (2005) Structural analysis of the anaphase-promoting complex reveals multiple active sites and insights into polyubiquitylation. *Mol Cell* 20, 855-866
5. Harper, J. W., Burton, J. L., and Solomon, M. J. (2002) The anaphase-promoting complex: it's not just for mitosis any more. *Genes & development* 16, 2179-2206
6. Shirayama, M., Zachariae, W., Ciosk, R., and Nasmyth, K. (1998) The Polo-like kinase Cdc5p and the WD-repeat protein Cdc20p/fizzy are regulators and substrates of the anaphase promoting complex in Saccharomyces cerevisiae. *The EMBO journal* 17, 1336-1349
7. Nagai, M., and Ushimaru, T. (2014) Cdh1 is an antagonist of the spindle assembly checkpoint. *Cellular signalling* 26, 2217-2222
8. Brownridge, P., Holman, S. W., Gaskell, S. J., Grant, C. M., Harman, V. M., Hubbard, S. J., Lanthaler, K., Lawless, C., O'Cualain, R., Sims, P., Watkins, R., and Beynon, R. J. (2011) Global absolute quantification of a proteome: Challenges in the deployment of a QconCAT strategy. *Proteomics* 11, 2957-2970
9. Brownridge, P., Lawless, C., Payapilly, A. B., Lanthaler, K., Holman, S. W., Harman, V. M., Grant, C. M., Beynon, R. J., and Hubbard, S. J. (2013) Quantitative analysis of chaperone network throughput in budding yeast. *Proteomics* 13, 1276-1291
10. Lawless, C., and Hubbard, S. J. (2012) Prediction of missed proteolytic cleavages for the selection of surrogate peptides for quantitative proteomics. *Omics* 16, 449-456
11. Silva, J. C., Gorenstein, M. V., Li, G.-Z., Vissers, J. P. C., and Geromanos, S. J. (2006) Absolute Quantification of Proteins by LCMSE : A Virtue of Parallel ms Acquisition. *Molecular & Cellular Proteomics* 5, 144-156
12. Wiśniewski, J. R., Zougman, A., and Mann, M. (2009) Combination of FASP and StageTip-Based Fractionation Allows In-Depth Analysis of the Hippocampal Membrane Proteome. *Journal of Proteome Research* 8, 5674-5678
13. Rappsilber, J., Mann, M., and Ishihama, Y. (2007) Protocol for micro-purification, enrichment, pre-fractionation and storage of peptides for proteomics using StageTips. *Nat. Protocols* 2, 1896-1906
14. Cox, J., and Mann, M. (2008) MaxQuant enables high peptide identification rates, individualized p.p.b.-range mass accuracies and proteome-wide protein quantification. *Nat Biotech* 26, 1367-1372

15. Cox, J., Neuhauser, N., Michalski, A., Scheltema, R. A., Olsen, J. V., and Mann, M. (2011) Andromeda: A Peptide Search Engine Integrated into the MaxQuant Environment. *Journal of Proteome Research* 10, 1794-1805
16. Brownridge, P., and Beynon, R. J. (2011) The importance of the digest: proteolysis and absolute quantification in proteomics. *Methods* 54, 351-360
17. Arava, Y., Wang, Y., Storey, J. D., Liu, C. L., Brown, P. O., and Herschlag, D. (2003) Genome-wide analysis of mRNA translation profiles in Saccharomyces cerevisiae. *Proceedings of the National Academy of Sciences of the United States of America* 100, 3889-3894
18. Ingolia, N. T., Ghaemmaghami, S., Newman, J. R. S., and Weissman, J. S. (2009) Genome-Wide Analysis in Vivo of Translation with Nucleotide Resolution Using Ribosome Profiling. *Science* 324, 218-223
19. Kertesz, M., Wan, Y., Mazor, E., Rinn, J. L., Nutter, R. C., Chang, H. Y., and Segal, E. (2010) Genome-wide measurement of RNA secondary structure in yeast. *Nature* 467, 103-107
20. Subtelny, A. O., Eichhorn, S. W., Chen, G. R., Sive, H., and Bartel, D. P. (2014) Poly(A)-tail profiling reveals an embryonic switch in translational control. *Nature* 508, 66-71
21. dos Reis, M., Savva, R., and Wernisch, L. (2004) Solving the riddle of codon usage preferences: a test for translational selection. *Nucleic Acids Research* 32, 5036-5044
22. Costello, J., Castelli, L., Rowe, W., Kerhsaw, C., Talavera, D., Muhammad, S., Sims, P., Grant, C., Pavitt, G., Hubbard, S., and Ashe, M. (2015) Global mRNA selection mechanisms for translation initiation. *Genome Biol* 16, 10
23. Vogel, C., Abreu Rde, S., Ko, D., Le, S. Y., Shapiro, B. A., Burns, S. C., Sandhu, D., Boutz, D. R., Marcotte, E. M., and Penalva, L. O. (2010) Sequence signatures and mRNA concentration can explain two-thirds of protein abundance variation in a human cell line. *Molecular systems biology* 6, 400
24. Zur, H., and Tuller, T. (2013) Transcript features alone enable accurate prediction and understanding of gene expression in S. cerevisiae. *BMC Bioinformatics* 14, S1
25. Geisberg, Joseph V., Moqtaderi, Z., Fan, X., Ozsolak, F., and Struhl, K. (2014) Global Analysis of mRNA Isoform Half-Lives Reveals Stabilizing and Destabilizing Elements in Yeast. *Cell* 156, 812-824
26. Belle, A., Tanay, A., Bitincka, L., Shamir, R., and O'Shea, E. K. (2006) Quantification of protein half-lives in the budding yeast proteome. *Proceedings of the National Academy of Sciences of the United States of America* 103, 13004-13009
27. Rechsteiner, M., and Rogers, S. W. (1996) PEST sequences and regulation by proteolysis. *Trends in Biochemical Sciences* 21, 267-271
28. Csárdi, G., Franks, A., Choi, D. S., Airoldi, E. M., and Drummond, D. A. (2015) Accounting for Experimental Noise Reveals That mRNA Levels, Amplified by Post-Transcriptional Processes, Largely Determine Steady-State Protein Levels in Yeast. *PLoS Genet* 11, e1005206
29. Wang, Y., Liu, C. L., Storey, J. D., Tibshirani, R. J., Herschlag, D., and Brown, P. O. (2002) Precision and functional specificity in mRNA decay. *Proceedings of the National Academy of Sciences* 99, 5860-5865
30. Gerashchenko, M. V., Lobanov, A. V., and Gladyshev, V. N. (2012) Genome-wide ribosome profiling reveals complex translational regulation in response to oxidative stress. *Proceedings of the National Academy of Sciences* 109, 17394-17399

31. Nagalakshmi, U., Wang, Z., Waern, K., Shou, C., Raha, D., Gerstein, M., and Snyder, M. (2008) The Transcriptional Landscape of the Yeast Genome Defined by RNA Sequencing. *Science* 320, 1344-1349
32. Holstege, F. C. P., Jennings, E. G., Wyrick, J. J., Lee, T. I., Hengartner, C. J., Green, M. R., Golub, T. R., Lander, E. S., and Young, R. A. (1998) Dissecting the Regulatory Circuitry of a Eukaryotic Genome. *Cell* 95, 717-728
33. Wang, M., Weiss, M., Simonovic, M., Haertinger, G., Schrimpf, S. P., Hengartner, M. O., and von Mering, C. (2012) PaxDb, a database of protein abundance averages across all three domains of life. *Mol Cell Proteomics* 11, 492-500
34. Lu, P., Vogel, C., Wang, R., Yao, X., and Marcotte, E. M. (2007) Absolute protein expression profiling estimates the relative contributions of transcriptional and translational regulation. *Nat Biotechnol* 25, 117-124
35. Newman, J. R., Ghaemmaghami, S., Ihmels, J., Breslow, D. K., Noble, M., DeRisi, J. L., and Weissman, J. S. (2006) Single-cell proteomic analysis of S. cerevisiae reveals the architecture of biological noise. *Nature* 441, 840-846
36. de Godoy, L. M., Olsen, J. V., Cox, J., Nielsen, M. L., Hubner, N. C., Frohlich, F., Walther, T. C., and Mann, M. (2008) Comprehensive mass-spectrometry-based proteome quantification of haploid versus diploid yeast. *Nature* 455, 1251-1254
37. Ghaemmaghami, S., Huh, W. K., Bower, K., Howson, R. W., Belle, A., Dephoure, N., O'Shea, E. K., and Weissman, J. S. (2003) Global analysis of protein expression in yeast. *Nature* 425, 737-741
